# The effectiveness of interventions during the first 1,000 days to improve energy balance‐related behaviors or prevent overweight/obesity in children from socio‐economically disadvantaged families of high‐income countries: a systematic review

**DOI:** 10.1111/obr.13524

**Published:** 2022-11-17

**Authors:** Sandrine Lioret, Faryal Harrar, Delia Boccia, Kylie D. Hesketh, Konsita Kuswara, Céline Van Baaren, Silvia Maritano, Marie‐Aline Charles, Barbara Heude, Rachel Laws

**Affiliations:** ^1^ Université Paris Cité, INSERM, INRAE, CRESS Paris France; ^2^ Faculty of Public Health and Policy, Department of Global Health and Development London School of Hygiene and Tropical Medicine London UK; ^3^ Institute for Physical Activity and Nutrition, School of Exercise and Nutrition Science Deakin University Geelong Australia; ^4^ Department of Medical Sciences Università di Torino, Cancer Epidemiology Unit Turin Italy

**Keywords:** infancy, intervention, poverty, pregnancy

## Abstract

This narrative systematic review examined effectiveness of interventions during pregnancy and up to 2 years of age in improving energy balance‐related behaviors or prevent overweight/obesity in children from families experiencing socio‐economic disadvantage. We identified 24 interventions, from 33 articles, since 1990. Overall, despite their heterogeneity and variability in internal and external validity, there was some evidence of beneficial impact of interventions on obesity risk (4/15), and associated behaviors, e.g.: breastfeeding (9/18), responsive feeding (11/16), diet (7/8), sedentary (1/3) and movement (4/7) behaviors, and sleep (1/2). The most effective interventions aimed at promoting breastfeeding commenced antenatally; this was similar for the prevention of obesity, provided the intervention continued for at least 2 years postnatally and was multi‐behavioral. Effective interventions were more likely to target first‐time mothers and involve professional delivery agents, multidisciplinary teams and peer groups. Among ethnic/racial minorities, interventions delivered by lay agents had some impact on dietary behavior but not weight outcomes. Co‐creation with stakeholders, including parents, and adherence to theoretical frameworks were additional ingredients for more pragmatic, inclusive, non‐judgmental, and effective programs. The growing body of evidence on obesity prevention interventions targeting families experiencing socio‐economic disadvantage is promising for reducing early inequalities in obesity risk.

**Abbreviations**
BF: Breastfeeding
EBRBs: Energy balance‐related behaviors
OB: Obesity
OW: Overweight
RoB: Risk‐of‐bias
WIC: Women, Infants, and Children

## 1 INTRODUCTION

Overweight and obesity (OW/OB) have reached alarming rates worldwide, with 38.9 million children <5 years affected in 2021.^1^ More than 25% preschool children are impacted in Southern Europe (e.g. Greece, Italy, Portugal),^2^, ^3^, ^4^ United States of America (USA)^5^ and Australia.^6^ Such early and high prevalence suggests that risk factors are in play even earlier. The World Health Organization Commission on Ending Childhood Obesity highlighted the importance of addressing obesity risks in the “first 1000 days”, i.e. the period from conception to age 2 years.^7^ Early risk factors of child OW/OB and suboptimal growth include parental pre‐pregnancy OW/OB; gestational weight gain; smoking during pregnancy; macrosomia; rapid weight gain; suboptimal feeding practices, such as non‐responsive formula feeding, short breastfeeding (BF) duration, putting infants to bed with a bottle, early introduction of solids; and suboptimal energy balance‐related behaviors (EBRBs), such as energy‐dense and nutrient‐poor dietary intake, screen sedentary behaviors, low levels of physical activity and inadequate sleep.^4^, ^8^, ^9^, ^10^, ^11^, ^12^, ^13^

Although a plateauing of child OW/OB prevalence has been reported in several high‐income countries since the 2000s,^14^ it remains a major public health issue. Children with OW/OB are more likely to become adults with obesity.^4^, ^15^ Childhood OW/OB has a negative impact on physical and mental health,^4^, ^16^, ^17^ but also on human capital development, whether characterized by cognitive performance, educational attainment or labor market outcomes later in life.^18^ What is more, childhood OW/OB disproportionally affects populations experiencing socio‐economic disadvantage and ethnic/racial sub‐population groups,^4^, ^5^, ^19^, ^20^, ^21^, ^22^ with recent trends showing an increase in such social inequalities.^23^ Occurrences of OW/OB often co‐exist with food insecurity, the latter ranging 8–15% in high‐income countries in 2020,^24^ leading to the so‐called double burden of malnutrition.^4^, ^25^ Noteworthy, the above‐mentioned early risk factors are also socially patterned^26^, ^27^, ^28^ and partly mediate the inverse association between socio‐economic position of the parents and childhood OW/OB.^11^, ^29^, ^30^ Social inequalities are thus transmitted from one generation to the next, with health inequities starting from birth.^31^, ^32^

The first 1,000 days is an opportune time to support parents, as primary caregivers and role model, to promote a healthy lifestyle and prevent obesity for their children.^31^, ^33^, ^34^, ^35^ EBRBs are set early and tend to track over the lifecourse,^36^, ^37^ hence the importance of developing healthy behaviors from early life. Universal and individual‐based approaches have not been effective so far to sustainably change behaviors across the whole population and have rather increased social inequalities in health; however, the impact of interventions targeted to families experiencing socio‐economic disadvantage, supposedly more effective to tackle social inequities, have not yet been encapsulated.^4^, ^38^, ^39^, ^40^, ^41^, ^42^ A previous systematic review suggested modest but promising effects of few early obesity prevention interventions implemented in such contexts, while calling for further high quality studies and longer follow ups.^43^ Given these studies have doubled since its publication in 2014, we aimed to update the current evidence on the effectiveness of family‐based interventions implemented during pregnancy and up to 2 years of age to improve EBRBs and growth, or prevent OW/OB, in children growing up in families experiencing socio‐economic disadvantage.

## 2 METHODS

This systematic review followed the Preferred Reporting Items for Systematic reviews and Meta‐Analyzes (PRISMA)^44^, ^45^ (Supplementary document S1) and AMSTAR‐2^46^ guidelines; and has been registered on PROSPERO (Registration ID number CRD42020166483).

### 2.1 Study selection criteria

#### 2.1.1 Inclusion criteria

The review included (cluster) randomized controlled trials and quasi‐experimental studies; quasi‐experimental studies were deemed eligible because it is not always possible to conduct randomized controlled trials in deprived and hard‐to‐reach populations, but we included them only if a control group was defined. Only studies implemented in high‐income countries as defined by the World Bank were included, as differences in education systems, modes of delivery of interventions, socio‐cultural and contextual differences could affect the generalizability of the findings. The population, intervention, comparison, outcome and timeframe characteristics of the search strategy are detailed in Table 1 according to the PICOT framework.^44^, ^46^

**TABLE 1:** Characteristics of the search strategy according to the PICOT framework

Population	Parents experiencing socio‐economic disadvantage (during pregnancy or the first 2 years of the child life), i.e.: those identified with low socio‐economic position (e.g. measured by education, occupation, or income); those eligible for Special Supplemental Nutrition Program for Women's, Infants, and Children (WIC), Early Head Start programs, or Medicaid (USA); those experiencing psycho‐social risks (e.g. teen pregnancy, single motherhood, depression) or housing difficulties; or those living in disadvantaged areas.
Intervention	Interventions had to be delivered in the first 1,000 days (herein defined as pregnancy and the first two years of the child) with the aim of improving one or more of the outcomes described below. Interventions could be directed at individual behavior change (e.g. individual counselling, audio‐visual materials, social support) or include structural components (e.g. incentives, vouchers, food stamps, coupons to facilitate healthy behaviors; and referral to social and health support services in the community).
Comparison	Intervention studies had to include a control group, i.e. a group of parents/infants who were not exposed to the intervention or who received ‘usual care’.
Outcome(s)	Studies had to address one or more of the following outcomes in children: ‐ Parental feeding practices, such as breastfeeding (BF)^a^ and age at complementary feeding; ‐ Eating behaviors, dietary intake; ‐ Physical activity and movement measures, such as outdoor play and tummy time; ‐ Sedentary behaviors, such as screen time, TV viewing, and time spent restrained; ‐ Sleep^b^; ‐ Anthropometric or growth measures, such as weight, height, body mass index (BMI), OW, OB, percent body fat, skin fold thickness, weight gain velocity^c^.
Timeframe for follow‐up	Childhood: at least one of the eligible outcomes had to be assessed for effectiveness beyond the childbirth.

^a^ BF should however not be the only focus/outcome of the intervention; ^b^Sleep should however not be the only focus/outcome of the intervention;

^c^ Anthropometrics at birth should however not be the only outcome(s) assessed.

#### 2.1.2 Exclusion criteria

Studies self‐identified as pilot/feasibility studies and those with less than 30 participants in either intervention arm were not eligible. We also excluded those reporting on children with an average age of >24 months at the start of the intervention; targeting Indigenous populations (given uniqueness of their history and contexts); focused on ethnic/racial minorities without adding any inclusion criterion characterizing socio‐economic disadvantage; targeting children with a critical illness (including OW/OB) or eating disorder, or any disability influencing dietary intake, physical activity or sleep; and those focused on maternal health without clearly aiming to assess impact on outcomes in children too. We also excluded single behavioral interventions exclusively focused either on BF or sleep in children.

In all, the main distinguishing features of this systematic review, in comparison with the previous one published in 2014 by Laws et al,^43^ are the following: indigenous populations were excluded; interventions were required to target families experiencing socio‐economic disadvantage (in the review by Laws et al, studies of all populations were included if the findings were stratified by one or more socio‐economic indicator [e.g. education/income]); and interventions were required to be implemented prior to age 2 years (Laws et al included interventions up to 5 years of age).

### 2.2 Search

The following databases were searched: PubMed/MEDLINE, EMBASE, CINAHL, PsycINFO, and Scopus. Additionally, reference lists of included articles and relevant systematic reviews^43^, ^47^, ^48^, ^49^, ^50^, ^51^, ^52^ were cross‐checked, with any potentially eligible papers screened using standard methodology. Finally, experts in the field including the current authors (e.g. KDH & RL have authored previous systematic reviews on similar topics) and members of the LifeCycle Project‐EU Child Cohort Network within which this work was nested were consulted to identify any other papers that should be included in the screening. The search was performed 7th January 2022 and included articles from peer‐reviewed English language journals published since 1990 (inclusive). The search strategy is detailed in Supplementary document S2.

### 2.3 Study selection

**FIGURE 1:**
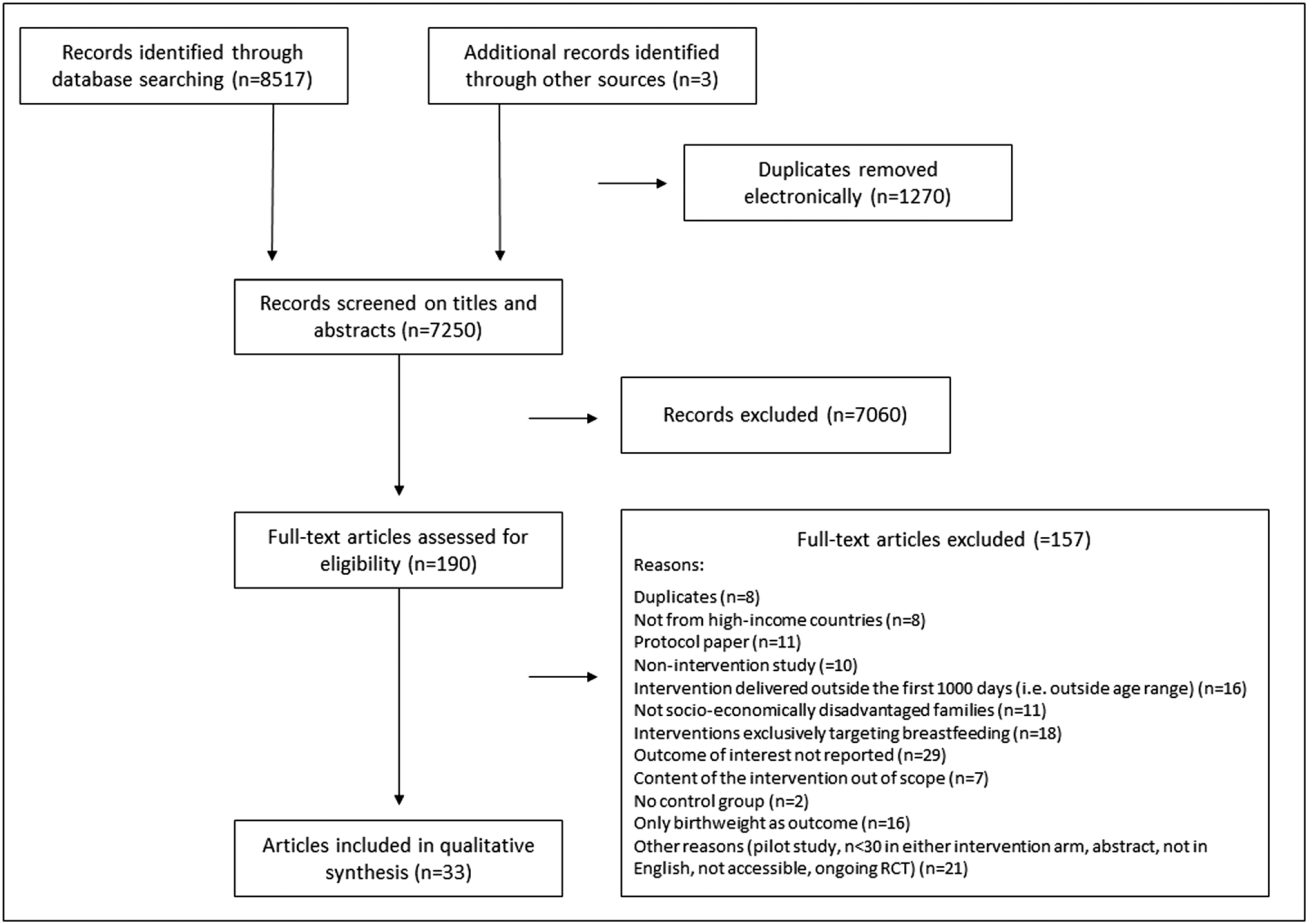
PRISMA flow chart resulting from the search strategy

The references retrieved from the database searches were transferred to *Covidence* (https://www.covidence.org) for eliminating duplicates and screening. Two authors (FH and SL) independently screened the titles and abstracts, then read full‐text articles of the remaining references to confirm their eligibility. During the whole process, summarized in a PRISMA chart^44^, ^45^ (Figure 1), any disagreement between FH and SL was flagged in *Covidence*, and solved through specific discussions, without needing a third reviewer.

### 2.4 Data extraction

An Excel template was used to extract data, which included the following fields: country, study design and methodology, enrolment and recruitment details, population and participant demographics, baseline characteristics, intervention details according to TIDieR guidelines,^53^ control conditions (if any), outcomes (measures and times of measurement) and measures of engagement, adherence and acceptability to users, retention in the study, results, and conclusions. All published articles and supplementary data related to the selected study, i.e. protocol paper; study registration website; pilot, qualitative, implementation and long‐term follow‐up studies; were considered for data extraction. SL cross‐checked the data extracted by FH, after reading all articles and supplementary material. Any disagreement or missing information in data extraction was resolved through discussion.

### 2.5 Quality assessment

The internal validity of studies was assessed for five domains (i.e. risk‐of‐bias (RoB) arising from the randomization process, RoB due to deviations from the intended interventions, missing outcome data, RoB in measurement of the outcome, and RoB in selection of the reported result), using the recently revised Cochrane RoB version 2 tool for randomized trials.^54^ For conciseness, outcomes were not assessed individually for RoB, but by themes, i.e. feeding practices, diet, physical activity, sedentary behaviors, sleep, and anthropometrics.

To assess the extent to which findings from studies could be generalized to populations or settings beyond the original study, a previously developed external validity assessment tool^55^ was used.^56^ The tool included five main components: 1) reach and representativeness (individuals); 2) reach and representativeness (settings); 3) implementation and adaptation (of intervention); 4) outcomes for decision makers; 5) maintenance and institutionalization. Institutionalization refers to the potential for implementation of the intervention in routine service delivery.^56^ Studies were coded according to whether they met each element (yes, no or not applicable).

Two authors (FH and SL) independently assessed internal and external validity of each study, and disagreements were resolved by consensus. Studies were not excluded from the review based on quality ratings.

Template data collection forms used in the review are available on request.

### 2.6 Synthesis of results

Given the heterogeneity of interventions, outcomes, measurements, and the age of children assessed, findings from the selected studies were synthesized narratively.

## 3 RESULTS

### 3.1 Identification of studies

The database searches yielded 8,517 records, of which 1,270 duplicates were removed. An additional 7,060 records were excluded after titles and abstracts screening, resulting in 190 full‐text articles to be read in full. A further 157 articles were excluded for various reasons detailed in the PRISMA flow chart (Figure 1). Overall, 33 studies (35 including two erratum articles) met the eligibility criteria, including 3 identified through reference lists.^57^, ^58^, ^59^, ^60^, ^61^, ^62^, ^63^, ^64^, ^65^, ^66^, ^67^, ^68^, ^69^, ^70^, ^71^, ^72^, ^73^, ^74^, ^75^, ^76^, ^77^, ^78^, ^79^, ^80^, ^81^, ^82^, ^83^, ^84^, ^85^, ^86^, ^87^, ^88^, ^89^, ^90^, ^91^ We extracted data from an additional 55 supplementary materials (other articles related to the intervention and protocol registration websites).

### 3.2 Study characteristics

The 33 included studies corresponded to 24 distinct interventions. The latter are summarized for their main characteristics in Tables 2, 3 and 4. Just over half of the interventions (n = 13; 54%) were published since the last review on a similar topic by Laws et al^43^ in 2014.

**TABLE 2:** Intervention characteristics (n = 24)

	Number (%)	Studies' references
**STUDY CHARACTERISTICS**		
**Publication date**		
1990–2014	11 (45.8)	^57^, ^69^, ^71^, ^72^
2014 and after	13 (54.2)	^70^, ^73^, ^91^
**Study design**		
(Cluster) randomized controlled trials	21 (87.5)	^57^, ^59^, ^61^, ^62^, ^64^, ^65^, ^67^, ^91^
Quasi‐experimental	3 (12.5)	^60^, ^63^, ^66^
**Previous pilot study**		
Yes	8 (33.3)	^63^, ^67^, ^69^, ^73^, ^76^, ^81^, ^83^, ^84^, ^85^
No	16 (66.6)	^57^, ^62^, ^64^, ^66^, ^70^, ^72^, ^77^, ^80^, ^82^, ^86^, ^91^
**Additional qualitative study**		
Yes	10 (41.7)	^60^, ^62^, ^67^, ^72^, ^74^, ^76^, ^81^
No	14 (58.3)	^57^, ^59^, ^63^, ^66^, ^73^, ^77^, ^80^, ^82^, ^91^
**Assessment of sustainability regarding outcomes eligible for the current review: additional follow‐up study/studies**		
Yes	5 (20.8)	^57^, ^58^, ^64^, ^65^, ^67^, ^69^, ^75^, ^76^, ^83^, ^84^
No	19 (79.2)	^59^, ^63^, ^66^, ^70^, ^74^, ^77^, ^82^, ^85^, ^91^
**POPULATION**		
**Country where the intervention was implemented**		
U.S.A	13 (54.2)	^59^, ^61^, ^63^, ^66^, ^72^, ^73^, ^77^, ^81^, ^83^, ^89^, ^91^
England	3 (12.5)	^62^, ^64^, ^65^, ^75^, ^76^
Australia	3 (12.5)	^67^, ^70^, ^90^
Republic of Ireland	2 (8.3)	^57^, ^58^, ^71^, ^82^
Northern Ireland	1 (4.2)	^71^
Netherlands	1 (4.2)	^74^
Chile	1 (4.2)	^60^
**Criteria used to screen socio‐economic disadvantage**		
Individually‐based, i.e.: income [1], employment [2], occupation [3], education level [4], eligible or enrolled in the WIC or Early Head Start programs [5], single motherhood [6], psycho‐social risk [7], housing difficulties [8], eligible to Medicaid [9], mother with OW/OB [10]	15 (62.5)	[>1 of criteria 1,2,4,6,7]^90^; [1,5]^61^, ^63^; [3]^64^, ^65^; [5]^66^, ^73^, ^91^; [9,10]^81^; [1]^86^, ^87^; [7]^70^; [6,7,or 8]^75^, ^76^; [≥2 of criteria 2,4,6]^59^; [1]^88^, ^89^; [criteria 4 and >1 of 1,2,7,8]^74^; [5,10]^83^, ^84^
Geographically‐based, i.e.: low socio‐economic level/deprived area, health center in such an area	9 (37.5)	^57^, ^58^, ^60^, ^62^, ^67^, ^69^, ^71^, ^72^, ^77^, ^80^, ^82^, ^85^
**Ethnic/racial minority group specifically targeted**		
Yes	6 (25.0)	^59^, ^61^, ^66^, ^77^, ^80^, ^83^, ^84^, ^88^, ^89^
No	18 (75.0)	^57^, ^58^, ^60^, ^62^, ^65^, ^67^, ^76^, ^81^, ^82^, ^85^, ^87^, ^90^, ^91^
**Ethnic/racial minority group representing >40% of the targeted population**		
Yes	17 (70.8)	^59^, ^61^, ^62^, ^64^, ^66^, ^70^, ^72^, ^81^, ^83^, ^89^, ^91^
No	7 (29.2)	^57^, ^58^, ^60^, ^63^, ^67^, ^69^, ^71^, ^82^, ^90^
**First‐time mothers**		
Yes	8 (33.3)	^57^, ^59^, ^61^, ^67^, ^69^, ^71^, ^74^, ^76^, ^85^
No	16 (66.7)	^60^, ^62^, ^66^, ^70^, ^72^, ^73^, ^77^, ^84^, ^86^, ^91^
**INTERVENTION**		
**Covering period**		
Pregnancy only	0	
Pregnancy + Post‐natal	15 (62.5)	^59^, ^67^, ^72^, ^74^, ^90^
Post‐natal only	9 (37.5)	^57^, ^58^, ^60^, ^66^, ^73^, ^91^
**Main setting**		
Home based	18 (75.0)	^57^, ^59^, ^61^, ^65^, ^67^, ^72^, ^74^, ^76^, ^82^, ^90^
Health care center, clinic, NGO	4 (16.7)	^60^, ^73^, ^77^, ^80^, ^91^
Video to watch at home	1 (4.2)	^66^
Social media	1 (4.2)	^81^
**Support type**		
One‐on‐one care	12 (50.0)	^57^, ^59^, ^61^, ^64^, ^65^, ^67^, ^69^, ^71^, ^74^, ^83^, ^87^
One‐on‐one care + group sessions	10 (41.7)	^60^, ^62^, ^63^, ^70^, ^75^, ^80^, ^82^, ^88^, ^91^
Digital support: social media or video to watch at home	2 (8.3)	^66^, ^81^
**Delivery agents** ^a^		
Paraprofessional agents or lay support: non‐professional peers, trained volunteers from the community, peer educators, community promotoras and doulas, etc.	11 (45.8)	^57^, ^58^, ^60^, ^61^, ^63^, ^65^, ^71^, ^72^, ^75^, ^76^, ^83^, ^84^, ^86^, ^89^
Nurses	6 (25.0)	^59^, ^67^, ^70^, ^74^, ^85^, ^90^
Dietician/nutritionist	2 (8.3)	^73^, ^77^, ^80^
Support health visitor, professional mentors	3 (12.5)	^62^, ^82^, ^91^
Psychologist	1 (4.2)	^81^
Video only	1 (4.2)	^66^
**Bilingual or community delivery agents**		
Yes	14 (58.3)	^57^, ^58^, ^60^, ^62^, ^64^, ^66^, ^71^, ^73^, ^75^, ^80^, ^83^, ^84^, ^86^, ^89^
No	10 (41.7)	^59^, ^63^, ^67^, ^70^, ^74^, ^81^, ^82^, ^85^, ^90^, ^91^
**Intervention intensity**		
Intensive	20 (88.3)	^57^, ^65^, ^67^, ^72^, ^74^, ^80^, ^82^, ^90^
Low intensity	4 (16.7)	^66^, ^71^, ^73^, ^81^
**Theory‐based intervention**		
Yes	18 (75.0)	^59^, ^61^, ^71^, ^74^, ^77^, ^82^, ^85^, ^91^
No	6 (25.0)	^57^, ^58^, ^60^, ^72^, ^73^, ^75^, ^76^, ^83^, ^84^
**Participatory approaches for program development**		
Yes	7 (29.2)	^61^, ^66^, ^73^, ^77^, ^84^
No	17 (70.8)	^57^, ^60^, ^62^, ^65^, ^67^, ^72^, ^74^, ^76^, ^85^, ^91^

Abbreviation: **NGO,** non‐governmental organization.

^a^Multidisciplinary teams: Alvarado et al^60^; Ordway et al^85^; Goldfeld et al^90^; Hans et al.^86^, ^87^

**TABLE 3:** For a given (set) of outcomes: number of interventions with either positive, negative or no effect (numerator), accounting for the number of interventions having assessed them (denominator)

	Energy balance‐related behaviors						Obesity risk indicators^a^	
	BF	PFP	Diet	PA	SB	SL	Birth: BW, BL	After birth: BMI, w/l
Positive effect (improvement)	9/18	11/16	7/8	4/7	1/3	1/2	0/7	2/6
Negative effect	0/18	0/16	0/8	0/7	0/3	0/2	1/7^b^	0/6
No significant effect	9/18	5/16	1/8	3/7	2/3	1/2	6/7	4/6

Abbreviations: **BF,** breastfeeding; **BL,** birth length; **BMI,** body mass index; **BW,** birth weight; **PA,** physical activity; **PFP,** Parental feeding practices other than BF; **SB,** sedentary behavior; **SL,** sleep; **w/l,** weight‐for‐length.

^a^ Other intervention effects based on either weight or height are presented in Table 4; ^b^Higher BW in the control group.

**TABLE 4:** Summary of interventions: target group, overarching aim, components, impact on outcomes at intervention conclusion, sample size at baseline, age at start and end, setting, delivery agent, follow‐ups, ethnic/minorities sub‐groups (if any)

	Components/Impact on outcomes assessed at intervention conclusion									N	Age at start/end	Setting/Delivery agent (recipient)	Follow‐ups	Ethnic/racial minority groups
Study/Target group/Intervention aim	BF	PFP	Diet	PA	SB	Anthro	SL	SM	TB					
Interventions primarily aimed at preventing OW/OB in children													
Wen et al (2011, 2012),^67^, ^68^, ^69^ Healthy Beginnings Trial Australia ‐ First time mothers living in disadvantaged areas of Sidney Aim: Prevent obesity by promoting healthy feeding practices and EBRBs	+	+	+	+	+	+BMI			☑	667	Preg/24 m	Home‐visiting/Trained nurses (one‐on‐one care)	6 m, 12 m, 24 m; 3y^a^, 5y^a^	Born overseas: 36%
Bonuck et al. (2014),^73^ The Feeding Young Children Study (FYCS) U.S.A ‐ Low income WIC attendees Aim: Prevent obesity by reducing inappropriate bottle feeding practices		+	+			øw/l				299	12 m/24 m	WIC center/nutritionist (one‐on‐one care)	12 m, 15 m, 18 m, 21 m, 24 m	Father and mother born abroad: 52% and 44%
Gross et al. (2016),^77^, ^78^, ^79^, ^80^ Starting Early Program (StEP) U.S.A ‐ Low‐income Hipanic/Latino families Aim: Prevent obesity by promoting healthy feeding practices and EBRBs	+	+	+	+	ø	+w/a			☑	533	Preg/3y	Health care center/Bilingual dietitians (one‐on‐one care + peer group sessions)	3 m, 10 m, 18 m, 24 m; 36 m	Hisp./Latina: 100%
Fiks et al. (2017),^81^ Grow2Gether U.S.A ‐ Low income Medicaid insured mothers with BMI > 25 kg/m^2^ Aim: Prevent obesity, promote healthy EBRBs, parenting and maternal well‐being	ø	+		ø	ø	øw/l	ø		☑	111	Preg/9 m	Psychologist (interactive Facebook peer group, with videos)	Birth, 2 m, 4 m, 6 m, 9 m	AA: 88%; Hisp.: 2%; White: 6%; Other: 7%
Reifsnider et al. (2018)^83^, ^84^ U.S.A ‐ Low income OW pregnant Mexican‐American women enrolled in WIC Aim: Prevent obesity by promoting healthy feeding practices and EBRBs	ø	ø				øw/l				174	Preg/24 m	Home‐visiting/Promotoras, Lactation consultant 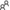 (one‐on‐one care)	36 wks (ga), 1wk, 1 m, 6 m, 12 m, 18 m; 24 m; 36 m^a^	Latina: 100%
Black et al. (2021)^91^ U.S.A ‐ Non‐pregnant low‐income mothers of toddlers Aim: Prevent obesity by promoting either healthy maternal lifestyle or responsive parenting interventions		+	+	+		øBMI			☑	277	20.1 m/24.1 m	WIC and pediatric centers/Health educators (peer groups + one‐on‐one care via phone calls)	6 m FU, 12 m FU	AA: 70%; Hisp.: 2%; non‐Hisp. White: 22%; Mixed/other: 6%
Interventions primarily aimed at promoting healthy feeding practices and diet													
Black et al. (2001)^61^ U.S.A ‐ Low‐income first‐time black adolescent mothers living with their mothers Aim: Delay introduction of complementary feeding		+							☑	121	4‐6wks/12 m	Home‐visiting/Trained black mentors 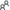 (one‐on‐one care)	3 m	AA: 100%
Horodynski et al (2005),^63^ Nutrition Education Aimed at Toddlers (NEAT) U.S.A ‐ Low income families enrolled in Early Head Starts EHS programs in rural Michigan counties Aim: Improve feeding practices		+							☑	135	1‐3Y/+ − 6 m	Home‐visiting/EHS home visitors & paraprofessional instructors 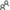 (peers) (one‐on‐one care + group sessions)	6 m	Not Caucasian: 16%
Watt et al. (2009),^64^, ^65^ Infant Feeding Peer Support Trial England ‐ Low income mothers living in culturally diverse and socially disadvantaged inner city London boroughs Aim: Improve feeding practices and child diet	ø	ø	+			‐W^b^, øL			☑	312	3 m/12 m	Home‐visiting/Trained community mothers 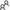 (one‐on‐one care)	12 m, 18 m; 4y^a^	50% self‐identified as ethnic minority groups
Scheinmann et al. (2010)^66^ U.S.A ‐ Immigrant Latina women attending WIC centers Aim: Improve feeding knowledge and practices	ø	+							☑	439	<5 m old/+6 m	Free 25‐min bilingual video	3 m, 6 m	Latina: 100%
Edwards et al. (2013)^72^ U.S.A ‐ Young pregnant low income women <22y Aim: Increase BF and improve PFP	+	+								248	Preg/3 m	Home‐visiting/Community Doulas 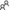 (one‐on‐one care)	Birth, 4 m	AA: 100%
Broad parent support programs														
Johnson et al. (1993),^57^, ^58^ Community mother's program Ireland ‐ First time low‐income mothers Aim: Improve child diet, health and development		+	+							262	Birth/12 m	Home‐visiting/Trained community mothers 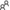 (one‐on‐one care)	12 m; 7y^a^	
Kitzman et al. (1997),^59^ Nurse–Family Partnership (NFP) U.S.A ‐ Low income AA first‐time mothers Aim: Improve maternal health and well‐being, birth outcomes and child development	+					øBW			☑	1,139	Preg/24 m	Home‐visiting/Trained nurses (one‐on‐one care)	28 wks, 36wks (ga), 6 m, 12 m, 24 m	AA: 92%
Alvarado et al. (1999)^60^ Chile ‐ Mothers living in disadvantaged neighborhood Aim: Improve maternal and infant nutrition and health	+					øBW,BL +W,H^c^				400	Birth/12 m	NGO/Trained health community workers 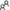 + NGO health pro staff (one‐on‐one care + group sessions)	Every month until 12 m	
Wiggins et al. (2005),^62^ Social support and family health (SSFH) study England ‐ Low‐income mothers living in culturally diverse and socially disadvantaged inner city London boroughs Aim: Improve feeding practices, and maternal and child health outcomes	ø	ø						ø	☑	731	10wks/+12 m	Home‐visiting/1.Support health home visitor; 2.Community groups (one‐on‐one care + group sessions)	12 m, 18 m	Self‐identified as black or minority ethnic group “non Whites”: 42%
Cupples et al. (2011),^71^ MOMENTS study Northern Ireland ‐ First‐time mothers living in socio‐economically deprived areas in Belfast Aim: Improve child growth, development and maternal health	ø			ø^d^		øBW,BL		ø	☑	343	Preg/12 m	Home visiting (& phone calls) /Trained peer‐mentor (one‐on‐one)	9 m, 12 m	
Kemp et al. (2011),^70^ Maternal Early Childhood Sustained Home‐visiting (MECSH) Australia ‐ At‐risk mothers, living in a socioeconomically disadvantaged suburb in Sydney Aim: Improve maternal health and well‐being and child health and development	+	ø		ø^d^		øBW		ø	☑	208	Preg/24 m	Home‐visiting/Trained Child and Family Health nurses (one‐on‐one care + possible parenting community groups)	1 m, 6 m, 12 m, 18 m, 24 m	Born overseas (31 different countries, but able to communicate in English): 49%
Medjoubi et al. (2014),^74^ VoorZorg programme Netherlands ‐ First time high risk pregnant women Aim: Reduce smoking, improve birth outcomes and child development and health	+					øBW		+	☑	460	Preg/24 m	Home‐visiting/Specialised VoorZorg nurses (one‐on‐one care)	16wks, 28wks, 32wks (ga), 2 m, 6 m	Surinamese/Antillean: 27%; Turkish 4%; Moroccan 2%; Cape Verdean 2%; Others: 16% (all understood Dutch language)
Kenyon et al. (2016),^75^, ^76^ Evaluation of Lay Support in Pregnant women with Social risk (ELSIPS) U.K ‐ Nulliparous mothers with social risk Aim: Improve maternal psychosocial health, maternal and neonatal birth outcomes, feeding practices and infant development	ø			+^d^, ^e^		øBW, W,H^f^				1,324	Preg/6wk	Home‐visiting/Specifically trained Pregnancy Outreach Workers 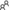 (one‐on‐one care, home, phone calls, groups)	6wks, 8‐12wks, 4 m; 12m^a^	Not Britain: 52%, including Asian: 26%; African: 7%; Caribbean: 5%; from Middle East: 3%
O'Sullivan et al (2017),^82^ Preparing for Life (PFL) Ireland ‐ Pregnant women living in socio‐economically disadvantaged communities Aim: Promote children's health, development and improve children's school readiness skills (here: improve child diet)			+						☑	233	Preg/5y	Home‐visiting/Professional mentors (one‐on‐one care + group sessions, when child aged 3y)	12 m, 18 m, 24 m, 36 m	
Ordway et al. (2018),^85^ Minding the Baby (MTB) U.S.A ‐ First time mothers from socio‐economically disadvantaged communities Aim: Develop and enhance parent–child attachment and reflective functioning and promote a range of positive parenting behaviors (here: improve child anthropometrics measures)	ø					‐BW^g^, +BMI			☑	237	Preg/24 m	Home‐visiting/Pediatric nurse and social worker (one‐on‐one care)	Birth, 12 m, 24 m	Hisp./Latina: 70%; AA: 22%
Hans et al. (2018)^86^, ^87^ U.S.A ‐ Young, low income families Aim: Improve birth outcomes, infant and maternal health and BF	+					øBW			☑	312	Preg/6wks	Home‐visiting/Community Doulas and Family Support Worker home visitors 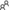 (one‐on‐one care)	37wks (ga), 3wks, 3 m	AA: 45%; Hisp./Latina: 38%; White: 8%; Multiracial/other: 9%
Lutenbacher et al. (2018),^88^, ^89^ The Maternal Infant Health Outreach Worker (MIHOW) program U.S.A ‐ Low income Hispanic women Aim: Improve maternal health and child development, feeding practices, combat isolation and increase access to health care	+	+							☑	188	Preg/6 m	Home‐visiting/Outreach workers = peer mentors 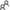 (one‐on‐one care + periodic group gatherings?)	35wks (ga), 2wks, 2 m, 6 m	Hisp.: 100%
Goldfeld et al. (2019),^90^ Right@home Australia ‐ Pregnant women experiencing psychosocial risks and living in socioeconomically disadvantaged regions in Victoria Aim: Improve feeding practices, child diet, sleep and safety; responsivity (parenting and bonding) and the home learning environment	ø	ø	ø				+	ø	☑	722	Preg/24 m	Home‐visiting/Nurse and social care practitioner (one‐on‐one care + group sessions)	6wk, 6 m, 12 m, 18 m, 24 m	

Abbreviations: **AA,** African American; **Anthro,** anthropometrics; **BF,** breastfeeding; **BL,** birth length; **BMI,** body mass index; **BW,** birth weight; **FU,** follow‐up; **ga,** gestational age; **Hisp.,** Hispanic; **Impact on outcomes,** + positive effect (improvement); − negative effect; ø no significant effect; **L,** length; **m,** months; **NGO,** non‐governmental organization; **PA,** physical activity; **PFP,** Parental feeding practices other than BF; **Preg,** pregnancy; **SB,** sedentary behavior; **SL,** sleep; **SM,** smoking; **TB,** theoretical basis of the intervention reported; III = , Intervention components (nor systematically assessed for effectiveness); **W,** weight; **w/l,** weight‐for‐length; **wk,** weeks; 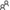, Lay support.

^a^ Follow‐up articles aimed at assessing sustainability

^b^ Weight was higher in the intervention group at 12 m

^c^ Higher weight and height at 6 and 12 months in the intervention group

^d^ Psychomotor development

^e^ Evaluation when infants were aged 12 m

^f^ Higher height and weight at 12 m

^g^ Higher BW in the control group.

#### 3.2.1 Design

Of the 24 interventions, nearly all were (cluster) randomized controlled trials; three had a quasi‐experimental design (Table 2). One third of the selected interventions were preceded by a pilot study (n = 8). Qualitative studies, based on semi‐structured interviews or observations, were further implemented for 42% of them (n = 10), either upstream, as a component of the pilot study^74^, ^92^, ^93^ or in the context of a participatory approach^61^; or downstream, to refine the process evaluation of the intervention.^60^, ^70^, ^72^, ^94^, ^95^, ^96^ All these qualitative studies were implemented among sub‐samples of mothers; and in fewer interventions, among delivery agents too (i.e. health care providers and lay workers).^60^, ^93^, ^95^, ^96^ Five follow‐up studies (21%) assessed sustainability of effectiveness beyond the end of the intervention.

#### 3.2.2 Population

A majority of the interventions were conducted in the USA (n = 13), seven in Europe, three in Australia and one in Chile (Table 2). Socio‐economic disadvantage was assessed based on individual characteristics in 63% of the studies (n = 15), such as: income; Women, Infants, and Children (WIC)/Early Head Start/Medicaid recipients; educational level; unemployment; psychosocial vulnerabilities; and housing difficulties. The remaining nine interventions were conducted in a deprived area, or a primary health care center in such an area, with no further confirmation of socio‐economic disadvantage assessed based on individual characteristics. In addition to socio‐economic disadvantage, ethnic or racial minority groups were specifically targeted in one quarter of the interventions (n = 6), thus composing 100% of the inclusion samples. Although not specifically targeted in 11 other interventions, ethnic or racial minorities represented >40% of the population included. Finally, while one third of the interventions targeted first‐time mothers (n = 8), none of them specifically targeted fathers/partners, but two did engage them in the support provided,^72^, ^74^ or other family members,^59^, ^72^ including the mother's mother.^61^

### 3.3 Interventions' characteristics

#### 3.3.1 Start and duration

We did not capture any antenatal‐only interventions with outcomes assessed for effectiveness in children beyond birth (Table 2). Nearly two thirds of the interventions started during pregnancy (most often during the third trimester) and were pursued post‐natally (n = 15). The two briefest interventions finished when the infant was aged 6 weeks^86^, ^87^ and 3 months,^72^ whereas Preparing for Life, the longest program, lasted until the child was aged 5 years^82^ (Table 4). Half of these ante‐ and post‐natal interventions lasted until toddlers were aged 24 months,^59^, ^67^, ^68^, ^69^, ^70^, ^74^, ^83^, ^85^, ^90^ and one until 36 months.^77^, ^78^ The remaining third are brief interventions implemented post‐natally only, with six of them in the first year of life^57^, ^60^, ^61^, ^62^, ^64^, ^66^ and the remaining three during the second year^63^, ^73^, ^91^ (Table 2).

#### 3.3.2 Setting, support type and delivery agent

Interventions were generally home‐based (n = 18) (Table 2). Only four were implemented at a primary health care center/clinic or an non‐governmental organization and another two were digitally supported, i.e. via a video to watch at home and social media. Half exclusively relied on one‐on‐one care, whereas 42% complemented this delivery mode with group sessions with peers (n = 10). About half of the programs were implemented based on lay (i.e. non‐professional) support (n = 11), most often with peers and doulas residing in the same community and trained to implement the program; the remaining interventions were most often delivered by nurses, then by dieticians/nutritionists, health visitors and professional mentors (n = 11). The Grow2Gether social media program was facilitated by a psychologist.^81^ Of all these programs, only four were conducted by multidisciplinary teams, i.e.: a nurse‐social worker^85^, ^90^ and a doula‐home visitor^86^, ^87^ dyads; or a pediatrician‐midwife‐social worker triad.^60^ In all, half of interventions were conducted with a special effort towards language diversity or socio‐cultural proximity, by the help of bilingual or lay/community delivery agents (n = 13).

#### 3.3.3 Components, intensity, theoretical framework, and consumer involvement

All 24 interventions had a component related to parental feeding practices or diet (Table 4). Additionally, six interventions promoted physical activity (or psychomotor development) or prevented sedentary behaviors in children. Sleep was also a theme for six of them, as was maternal smoking during pregnancy. Beyond individual counselling and social support, or referral to social support services at the community level, none of the interventions implemented any structural component, such as incentives, vouchers, food stamps, or coupons to facilitate healthy EBRBs (Table 2). All except four were intensive interventions, with a higher frequency of home visits or appointments than in the mainstream care system; and three quarters (n = 18) were theory‐based (Tables 2 and 4). Seven of these programs (protocols or tools) were developed based on a participatory approach, in co‐construction with recipients or community leaders (Table 2). In all, these 24 interventions can broadly be categorized into three types outlined below (Table 4).

Interventions (n = 6) primarily aimed at preventing the risk of OW/OB in children^67^, ^68^, ^69^, ^73^, ^77^, ^78^, ^79^, ^80^, ^81^, ^83^, ^84^, ^91^:

- four interventions started pre‐pregnancy, were multi‐component with themes encompassing various EBRBs, and rather intensive^68^, ^80^, ^81^, ^84^; the Feeding Young Children Study (FYCS)^73^ was implemented post‐partum and had a focus on parental feeding practices only; and the intervention by Black et al^91^ was multi‐behavior based too, but short (4‐month duration) and started later, at 20 months;
- four of them were theory based, i.e.: the Health Belief Model,^67^, ^68^, ^69^, ^77^, ^78^, ^79^, ^80^ the Social Cognitive theory,^77^, ^78^, ^79^, ^80^, ^81^ the Ecological theory,^77^, ^78^, ^79^, ^80^ and the Transactional theory^91^;
- the three interventions that more specifically targeted Hispanic/Latina women (USA) used a community‐based participatory approach with community leaders, WIC staff or clients to better adapt the programs' content and tools.^73^, ^77^, ^78^, ^79^, ^80^, ^83^, ^84^ Furthermore, delivery agents for these three programs were bilingual (Spanish/English);
- the intervention by Reifsnider et al^83^, ^84^ was delivered by trained community health workers (promotoras), whereas in the other five, delivery agents were health professionals.

Interventions (n = 5) primarily aimed at promoting healthy feeding practices and diet^61^, ^63^, ^64^, ^65^, ^66^, ^72^:

- all were aimed at enhancing parents' feeding knowledge and confidence and consisted in offering an empowering, practical and non‐judgmental support on infant feeding practices to the mothers; four were theory‐based, i.e.: the Ecological theory,^61^ the Support Theoretical Model,^64^, ^65^ the Social Cognitive Theory along with the Theory of Dependent Care,^63^ and anticipatory guidance^66^;
- the program by Scheinmann et al^66^ was low‐dose and relied on a 5‐minute video developed using professional production team and featuring WIC clients and locations; whereas the other four were based on home visiting and rather intensive, with two of them further supported by a videotape^61^ and peers' groups^63^;
- three trials specifically targeted African American^61^, ^72^ and Latina^66^ mothers in the USA and although the Infant Feeding Peer Support trial by Watt et al^64^, ^65^ in England did not focus on any particular ethnic group, 50% of the study population self‐identified as belonging to ethnic minorities. These four interventions relied on lay support and were implemented by trained community doulas or mothers.

Broad parent support programs (n = 13) with the overarching objective to enhance the lifestyle, general health and well‐being of the mother; the bonding/attachment with her child; infant care (including feeding and sleep); his/her development and general health^57^, ^58^, ^59^, ^60^, ^62^, ^70^, ^71^, ^74^, ^75^, ^76^, ^82^, ^85^, ^86^, ^87^, ^88^, ^89^, ^90^:

- most were inspired by the Elmira study and the Nurse Family Partnership (NFP) program^97^ and were underpinned by a variety of theoretical frameworks, such as the Listening Model of Support,^62^ the Ecological theory,^59^, ^70^, ^71^, ^85^ the Banduras' self‐efficacy theory,^59^ and the Attachment theory^59^;
- all except three^57^, ^60^, ^62^ commenced antenatally. They often started focusing on the pregnant woman, in listening to her requests and responding to her needs; offering regular parenting support and practical help to those who were under stress and experiencing difficulties; and helping to foster self‐confidence and links into other community services;
- all were intensive and essentially delivered through home‐visiting; nurses and health professionals were the delivery agents in seven of them,^59^, ^62^, ^70^, ^74^, ^82^, ^85^, ^90^ whereas lay support was preferred in the remaining six^57^, ^58^, ^60^, ^71^, ^75^, ^76^, ^86^, ^87^, ^88^, ^89^; group sessions with peers were further implemented in half of them^60^, ^62^, ^70^, ^75^, ^76^, ^82^, ^90^;
- all were selected in the current review because their authors secondary assessed effectiveness on various feeding practices (especially BF) or dietary outcomes, but also sleep,^90^ psychomotor development,^70^, ^71^, ^76^ anthropometric outcomes at birth^59^, ^60^, ^70^, ^74^, ^75^, ^85^, ^86^, ^87^ and after birth.^60^, ^71^, ^76^, ^85^

### 3.4 Impact on outcomes

Given the heterogeneity in outcomes and time points assessed it was not possible to synthesize the findings quantitatively. Anthropometric outcomes were favorably impacted in 4 out of 15 studies^60^, ^68^, ^80^, ^85^; BF (initiation and duration) in 9/18^59^, ^60^, ^67^, ^70^, ^72^, ^74^, ^77^, ^79^, ^86^, ^87^, ^88^, ^89^; other feeding practices (mostly age at complementary feeding, bottle feeding practices, and responsive feeding practices) in 11/16^57^, ^61^, ^63^, ^66^, ^67^, ^68^, ^72^, ^73^, ^77^, ^81^, ^88^, ^89^, ^91^; dietary intakes (assessed as food groups and nutrients) in 7/8^57^, ^58^, ^67^, ^68^, ^73^, ^79^, ^82^, ^91^; physical activity (including tummy time and psychomotor development) in 4/7^67^, ^76^, ^78^, ^91^; sedentary behavior (including exposure to screens and time restrained) in 1/3^68^; and sleep (including duration and routines) in 1/2^90^ (Tables 3 and 4).

Of the 15 interventions that reported anthropometric outcomes (Table 4), four measured them solely at birth,^59^, ^70^, ^74^, ^86^, ^87^ which were broad parent support programs; none resulted in any improvement of such anthropometric outcomes. Conversely, the majority of the 11 studies that compared anthropometric measures between intervention and control groups beyond birth were aimed at preventing OW/OB in children and were based on multi‐behavioral programs that focused on various feeding practices and EBRBs. Four of these interventions effectively impacted the risk of OW/OB or improved growth: in the Healthy Beginning Trial there was a difference of −0.29 kg.m^2^ between intervention and control arms at intervention conclusion, i.e. 24 months^68^; in the Starting Early Program (StEP), mean weight‐for‐age z‐scores and growth trajectories were lower for the intervention group through age 2 years^80^; although proportionally more children in Minding The Baby (MTB) program were in the OW category (16.3 versus 13.6% in the control arm) at 2 years, prevalence of children with OB was lower (3.3% versus 19.7%)^85^; and the quasi‐experimental trial by Alvarado et al^60^ showed greater weight and length at both 6 and 12 months. Compared to the seven programs with null results regarding anthropometrics, these four interventions had the following characteristics:

- they were more likely to start antenatally and to be of longer duration (until 12–36 months post‐partum);
- they were more likely to target first‐time mothers; they however did not specifically target nor reach ethnic/racial minorities;
- they were more likely to be underpinned by a behavior change theory, but less often preceded by a pilot study;
- all four were also effective in promoting healthy feeding practices and EBRBs, except the one by Ordway et al^85^;
- a variety of delivery agents implemented these four programs, but professionals, multidisciplinary teams and groups of peers were more often involved.

All programs that succeeded in promoting BF started antenatally (Table 4), and were more likely to incorporate lay support than those that did not have any impact on BF practices. Interventions that were effective in changing other feeding practices and dietary outcomes more often aimed to prevent OW/OB, involved consumers in their development, targeted first‐time mothers, and relied on lay support or a dietician/nutritionist, as compared to those that were not. Overall, interventions impactful on physical activity and sedentary outcomes shared common characteristics with those succeeding to reduce the risk of OW/OB. Lastly, impact on sleep was measured in two interventions only,^81^, ^90^ not allowing us to draw insightful conclusions. Still, the one by Fiks et al^81^ showed some benefit on sleep routines and duration: participatory approaches to the development of videos, interactive social media peer group, theory‐based and multi‐behavioral components were some of the notable features of this intervention.

Interventions assessed for sustainability 1 to 7 years later (n = 5 studies) revealed that former impacts on anthropometric outcomes at intervention conclusion (if any) were not maintained, nor improved, at the next follow‐ups.^58^, ^65^, ^69^, ^76^, ^84^ Better sustainability was however observed for feedings practices and EBRBs.^58^, ^65^

Although not in the scope of the current review, it is noteworthy that some of the broad parent support programs had a positive impact on various aspects of childbirth preparation (e.g., epidural/pain medication during labor),^86^, ^87^ the mother‐to‐infant bonding,^75^, ^76^ safety of new‐born care practices,^86^, ^87^, ^89^ the child health and development (e.g. injuries, immunizations, language, play cognitive games)^57^, ^58^, ^59^, ^88^, ^89^, ^90^; and the mother's knowledge on feeding practices,^63^, ^66^, ^77^, ^79^ health (e.g. tiredness, depression, self‐esteem and contraceptive practices),^57^, ^58^, ^59^, ^60^, ^75^, ^76^, ^81^, ^88^, ^89^, ^90^ diet,^57^, ^58^, ^68^ physical activity,^68^, ^91^ and smoking habits.^74^ Smoking was however not improved in most studies targeting it.^62^, ^70^, ^71^, ^90^

### 3.5 Internal validity

Most studies were rated as “low” or “some concerns” on Domains 1 (randomization), 4 (outcome measurement) and 5 (reporting of results), with a small number rated as “high RoB” (Table S1). Likewise, studies were predominantly rated as “low” or “some concerns” for Domain 3 (missing outcome data), with none rated as high RoB. Domain 2 (deviations from the intended interventions) was systematically rated as “some concerns”, given that it was inevitable that agents delivering the various interventions were aware of participants' assignment during the trials. Additionally, participants were aware of their assigned intervention, with the exception of one trial.^83^ Overall, 15 studies had at least one outcome assessed as low RoB in 4 domains out of 5,^62^, ^64^, ^67^, ^68^, ^69^, ^70^, ^71^, ^75^, ^80^, ^82^, ^86^, ^87^, ^90^ or 3 domains out of 5,^59^, ^70^, ^72^, ^74^, ^77^, ^78^, ^79^, ^81^, ^83^, ^84^, ^91^ reflecting a relatively good internal validity for that particular outcomes. Nearly all those that were rated with a high overall RoB did not have a strict randomized design.^60^, ^63^, ^66^, ^85^

### 3.6 External validity

#### 3.6.1 Reach and representativeness of individuals and settings (Tables S2a and S2b)

The target population and recruit methods (n = 23), inclusion and exclusion criteria (n = 20), and both the enrolment (n = 20) and recruitment/participation (n = 19) rates, were on the whole well described. Except for one study,^83^ participation rates were >50%, and ranged 39% to 97%. Far fewer studies (n = 6) described the representativeness of participants. In general, the target setting was described (n = 23), contrary to the other characteristics i.e.: methods to recruit it (n = 3), setting inclusion and exclusion criteria (n = 2), setting participation rate (n = 0) and representativeness of settings (n = 0).

#### 3.6.2 Implementation and adaptation (Table S2c)

Intervention characteristics (n = 23), actual exposure to the intervention (n = 19), delivery agents (n = 23) along with their training (n = 19), were on the whole well reported. This was also the case, though to a lesser extent (n = 15), for the time to deliver the intervention and methods to recruit delivery agents. However, delivery agent's participation rate (n = 2), intervention fidelity (n = 8), and mechanisms for intervention effects (n = 2), were relatively poorly reported.

#### 3.6.3 Outcomes for decision makers, maintenance and institutionalization (Table S2d)

Outcomes were always comparable to standards. Retention rates were well described (n = 23, ranging 45% to 96% at intervention conclusion), however representativeness of completers versus drop‐outs was reported in only 10 interventions. Information about acceptability of the intervention by stakeholders was provided for the majority of interventions (n = 15). External validity was however suboptimal in terms of adverse consequences reported (n = 3), moderation analyzes (n = 8), dose–response analyzes (n = 5), information on cost/cost‐effectiveness (n = 7), sustainability (n = 5) and institutionalization (n = 9).

## 4 DISCUSSION

This systematic review provides updated insights into a growing body of evidence on the impact of interventions implemented over the first 1,000 days to promote healthy feeding practices and EBRBs, or prevent OW/OB, among children growing up in families experiencing socio‐economic disadvantage. We found 24 distinct interventions, reported in 33 articles dating from 1990 to January 2022, which can be classified into three types, i.e.: those specifically aimed at preventing OW/OB in children (n = 6); those mostly focused on promoting healthy feeding practices and diet (n = 5); and broad parent support programs aimed at enhancing the general health and bonding of the mother–child dyad (n = 13). The majority were published since Laws et al review in 2014.^43^ These more recent interventions were more often started antenatally and continued post‐partum, theory‐based and developed with stakeholders and recipients (participatory research). Overall, despite the heterogeneity and large variability regarding internal and external validity of studies, there is some evidence of beneficial impacts of these interventions on the risk of OW/OB, as well as its associated behavioral factors. Ingredients of effective interventions depended on the populations targeted (e.g. mainstream population versus racial/ethnic minority groups, first‐time versus multiparous mothers), and the intervention characteristics, such as the engagement of stakeholders (including recipients) in their development; their start and duration; the components and theoretical frameworks involved; their setting; and delivery mode as discussed below.

### 4.1 Population

#### 4.1.1 Ethnic/racial minority groups

Even though interventions implemented among ethnic/racial minority groups experiencing disadvantage were overall less often effective, outreach seemed improved when delivery agents were bilingual (or helped with interpreters) and program tools translated, simplified and illustrated.^62^, ^64^, ^66^, ^73^, ^77^, ^83^, ^86^, ^87^, ^89^ Co‐designing these tools with recipients (e.g. booklets or videos) was also suggested to improve their uptake.^61^, ^66^, ^73^, ^77^, ^83^ Participatory approaches were essentially used to elaborate programs when such culturally and linguistically diverse populations were targeted, with improved outcomes, in particular feeding practices and diet.

#### 4.1.2 First‐time mothers

The transition into motherhood is a time when mothers/parents are usually more inclined to seek and receive advice regarding feeding and child rearing,^36^, ^47^, ^98^, ^99^ which could partly explain the greater effectiveness of programs targeting first‐time mothers. Whereas multiparous mothers are more exposed to socio‐economic constraints and food insecurity, they are supposedly less likely to seek support when experience is already acquired with previous babies, or because of higher time constraints given the presence of older siblings in the household. However, another advantage, or ripple effect, of supporting fist‐time mothers in their feeding practices and general lifestyle, stands on the fact that any subsequent pregnancy could further benefit from the former intervention. Noteworthy, fathers/partners, as recipients of the intervention, or for their involvement alongside mothers, were dramatically under‐represented in the studies reviewed here.

### 4.2 Intervention characteristics

#### 4.2.1 Start and duration

This review confirmed that a common trait of successful interventions for breastfeeding promotion and OW/OB prevention was that they commenced in pregnancy.^43^, ^51^, ^100^, ^101^, ^102^, ^103^ Decisions about infant feeding are often made during pregnancy.^104^, ^105^ Hence, tailored support during pregnancy is vital to encourage best practice infant feeding methods, especially when the mother faces a variety of adversities, inherent to social disadvantage, e.g.: psycho‐social vulnerabilities, lack of confidence in milk supply, and a lack of transmission by the family or peers due to social isolation.^103^, ^106^, ^107^, ^108^, ^109^, ^110^ Regardless of social disadvantage, other common barriers include a lack of knowledge or experience on such feeding practices and BF complications arising while initiating breastfeeding (e.g. mastitis and breast abscess). Empowering the woman through a personalized support during this transition to motherhood, anticipating the stages to come (anticipatory guidance), and engaging the father/partner to support the mother, is therefore essential to help her accomplish her goals. This is all the more important given the health (for mother and child), psycho‐social and economic benefits associated with BF.^28^, ^111^

In addition, interventions starting during pregnancy often had a strong focus on the pregnant woman's own health and lifestyle. Improving such maternal factors is beneficial for her well‐being and self‐efficacy and for the mother–child bonding after delivery, and may also help a modelling of dietary behaviors, screen use and movement/physical activity for the child.^33^, ^51^, ^112^, ^113^, ^114^, ^115^ The fact that these EBRBs are learned and formed early in life, track across childhood, and are involved in the development of adiposity, may partly explain why interventions starting antenatally and continued for >2 years after delivery are more effective for the prevention of childhood OW/OB.^36^, ^47^, ^52^, ^103^, ^115^

#### 4.2.2 Intervention components

Interventions that mostly focused on promoting healthy feeding practices/diet and broad parent support programs did not tend to focus on growth nor OW/OB. Conversely, the six interventions specifically aimed at preventing OW/OB in children seemed the most successful in doing so. These were more likely intensive, theory‐based and more often targeted multiple EBRBs. Of note, successful OW/OB prevention interventions were more often successful in changing other EBRBs too, such as BF and other feeding practices,^60^, ^67^, ^77^, ^79^ dietary intake,^68^, ^79^ screen exposure,^68^ and physical activity.^67^, ^78^ It can be hypothesized these factors played a mediating role in the effectiveness of the intervention, but this has not been specifically explored in any of these studies. Such multi‐behavioral interventions are potentiated by both the correlation and covariation of behaviors: effective change on one targeted behavior increases the probability of effective change on a second targeted behavior, thus synergistically contributing to greater impact of such complex interventions on health.^116^, ^117^, ^118^ This is all the more important as, depending on the social adversities encountered by the family, their specific barriers and facilitators, some behaviors are likely more amenable to change than others.

However, despite the known influence of smoking during pregnancy on the risk of childhood OW/OB, none of these six interventions aimed at preventing OW/OB had an additional focus on smoking prevention. Rates of smoking are usually higher in pregnant women experiencing disadvantage, with the opposite observed for BF practices.^27^, ^28^, ^119^ Furthermore, BF is rather contraindicated in smoking mothers, because the presence of nicotine and other contaminants from smoking in the breast milk. Therefore, supporting pregnant women to diminish (or stop) smoking, along with the promotion of BF and healthy EBRBs, would probably enhance the efficacy of childhood OW/OB prevention actions implemented in the first 1,000 days, while reducing social inequalities in health for the mother–child dyad.^49^, ^120^, ^121^ Whereas smoking was a target of six of the broad parent support programs started antenatally,^59^, ^62^, ^70^, ^71^, ^74^, ^90^ only one of them assessed the impact on anthropometric outcomes beyond birth, with no difference reported between intervention and control groups at 12 months.^71^ We cannot exclude that the other five had some unmeasured impact on OW/OB risk later in childhood.

#### 4.2.3 Individual versus structural intervention components and theoretical frameworks

Education and counselling at the individual level featured in all 24 interventions. Few of these interventions were grounded within the socioecological theoretical framework.^59^, ^61^, ^70^, ^71^, ^74^, ^82^, ^85^ However, beyond the links to other community social and health support services that were mainly set up within the broad parent support programs (e.g. for housing, ensuring benefits, employment, parenting, childcare, engagement with antenatal services), none of the programs reviewed here included nor evaluated other types of structural components to facilitate change in individual EBRBs. Yet it is now well known that interventions involving individual agency rather than structural changes at different levels of the socio‐ecological model, tend to increase socio‐economic inequalities in health.^41^ In fact, they most often fail to account for the specific socio‐economic, socio‐cultural, and socio‐demographic determinants of individual health behaviors faced by families living in underprivileged contexts.^4^, ^41^, ^49^, ^101^, ^103^, ^122^ For example, the observational study by Chaparro et al suggested that improving availability, accessibility and affordability to a larger range of healthy foods via the Special Supplemental Nutrition Program for Women, Infants, and Children (WIC) program was an important facilitator of individual dietary change and the prevention of childhood OW/OB.^123^, ^124^ These incentive or voucher‐style programs are all the more important given that food insecurity has dramatically increased worldwide since the COVID pandemic.^1^, ^4^, ^125^ Limiting further increases in socio‐economic inequities in OW/OB risk, and more generally health, from early life therefore requires the addition of structural components into family‐based interventions^41^, ^101^, ^103^; for example, vouchers/incentives to facilitate access to products and services are likely interesting options to enable healthy EBRBs, so as ‐ at the community level, urban design to increase green spaces in deprived neighborhoods, and regulation of density of fast foods restaurants versus greengrocer shops.^4^

#### 4.2.4 Settings and delivery agents

The current review confirmed that home‐visiting was relevant to reach families experiencing disadvantage, more often socially isolated and known to have a suboptimal use of health and community services.^43^, ^49^, ^51^, ^52^, ^115^ Beyond information provision, such one‐on‐one and face‐to‐face support allows for building a trusting relationship between the delivery agent and the mother, and for tailoring the intervention to her specific needs. On the one hand, nearly all home‐based interventions included in the review were intensive, scarcely assessed for their cost‐effectiveness, nor for representativeness of settings and delivery agents, which raise the question of their external validity in terms of transferability and scaling‐up into the real world. On the other hand, the rare low‐dose interventions based on films, videos or social media, without any face‐to‐face support, were not sufficient to change the outcomes of interest.

Health professionals appeared to be relevant delivery agents to promote responsive and healthy feeding practices and diet, which were a focus in all interventions. However, whatever the setting and outcomes assessed, effectiveness seemed enhanced when a multidisciplinary team was engaged, such as a social worker, in addition to a health professional. Social disadvantage encompasses a variety of individual profiles and it is noteworthy that the broad parent support programs had a more comprehensive approach of the general physical and psycho‐social health of the mother–child dyad, resulting in a wide range of foci beyond feeding practices and EBRBs. They often addressed the needs spontaneously expressed by mothers, as diverse as food insecurity, housing instability, physical abuse, discrimination, anxiety, to name but a few. Therefore, a multidisciplinary support is likely more amenable to both identify the diversity of risks and barriers encountered by mothers and envisage specific facilitators for their empowerment within such an adverse environment.^115^ Often underpinned by the social cognitive theory,^126^ program delivery via groups of peers, in addition to one‐on‐one support (whatever its setting), stands as an interesting complement to foster social support, sharing of experiences and strategies, skill‐building, health literacy, and interactions with peers.

Lay support by non‐professional trained workers characterized the majority of interventions delivered among ethnic/racial minority groups and was particularly promising for promoting healthy feeding practices, diet and motor skills. In addition to sharing a common language and socio‐cultural norms, this delivery mode reduces the hierarchical gap between the knower and the recipient, positioning the lay agent at a relatively equal level with the mother/parents. Peer‐mentorship more often involves friendship and high level of practical support, with this type of interaction perceived as less prescriptive, blaming and stigmatizing.^95^ Trust is also facilitated by their respectful and non‐judgmental approach. However, home visiting based on lay support alone, did not seem enough to influence other EBRBs nor the risk of OW/OB,^71^ and was optimized by incorporating sessions with group of peers or complementing the support with the specific competencies of a professional delivery agent.^60^, ^89^

In line with this, in other contexts (general population), it has been reported that interventions were more effective on BF outcomes when implemented in a combination of settings (health systems, home, community).^127^ Pregnancy and care of the newborn are opportunities for all families to engage with health‐care centers and their practitioners.^31^, ^49^ Utilizing these health care services in combination with existing home visitation programs, digitally based interventions, and community resources, so as to tailor and optimize primary prevention amongst the most at‐risk mother–child dyads, is likely a relevant avenue for scalability, sustainability and cost‐effectiveness of public health actions.^43^, ^115^

#### 4.2.5 Measurements

For an easier comparison between intervention studies and to enable a quantitative synthesis, there is a need to harmonize the core set of outcomes to be evaluated in future such interventions.^128^, ^129^ Of note, BMI, weight‐for‐height and weight‐for‐age z‐sores, the most common indicators used in these studies, are only proxies for measuring adiposity and the risk of OB, as they do not allow distinguishing between lean and fat masses. Such measurements are likely not sensitive enough to detect intervention effects.^102^ Additionally, weight, height and BMI curves or kinetics over these early years are likely more relevant to compare the dynamics of growth, in response to a given program, between intervention and control groups. For example, rapid weight gain was used to measure effectiveness of such early OB prevention interventions in other population groups.^51^ It is striking that none of the reviewed interventions compared any of the catch‐up growth, nor percent body fat, skin fold thickness, or waist circumference between intervention and control groups.

#### 4.2.6 Sustainability

Socially disadvantaged populations are known to be hard‐to‐survey, which not only means hard‐to‐sample, but also hard‐to‐identify, hard‐to‐reach, hard‐to‐persuade, hard‐to‐interview, and hard‐to‐follow‐up.^130^ In fact, attrition was variable but relatively high in some of the follow‐up studies reviewed here, ranging 21%^69^ to 92%^76^ (results not shown); and socio‐economically patterned in two of the three follow‐up studies that characterized factors associated with attrition.^69^, ^76^ This may have resulted in insufficient power and accuracy of the sample (no longer representative of the population at inclusion) to detect long‐lasting effects on OW/OB risk indicators. However and importantly, better sustainability was observed in the current review for intermediate behavioral risk factors. We cannot exclude that the interventions may have had later favorable, but unmeasured, impact on OW/OB, given that EBRBs tend to track from early life, into later childhood and adulthood.^43^, ^49^

### 4.3 Limitations and strengths

The scope of this review was broad, encompassing heterogeneous programs, foci, timeframes and outcomes, precluding any quantitative synthesis of findings. Still, the search from five different databases over the last 30 years, the thorough data extraction, the comprehensive internal and external validity assessment of the individual studies included, and their narrative synthesis, provide important insights into ingredients and processes that seem valuable to consider in future interventions aimed at tackling social inequalities in perinatal health. The registration into PROSPERO prior to the screening and adherence to PRISMA and AMSTAR‐2 guidelines reinforced the rigorousness and quality of the analysis undertaken. It is important to recognize that the grey literature was not addressed nor the evidence reported in non‐English publications. Additionally, publication bias cannot be excluded, with a possible over‐representation of “positive” interventions in the peer‐reviewed literature screened. However, several studies reported null results for some of the outcomes assessed for effectiveness, suggesting that at least some research is published regardless of its non‐significant findings.

### 4.4 Recommendations for practice and policy

Some ingredients of promising interventions targeting families experiencing socio‐economic disadvantage undertaken during the first 1,000 days' window of opportunity deserve to be highlighted for their relative effectiveness:

- recipient and stakeholder involvement in the development of actions through bottom‐up initiatives;
- multi‐behavioral programs, commencing in pregnancy, and continued at least two‐year post‐partum. Multifaceted interventions should focus not only on the mother/father lifestyle and well‐being, but also on parental feeding practices, and all EBRBs from infancy;
- promising strategies with regards to the mother's self‐confidence, self‐efficacy, and skills; the bonding to her child; responsive feeding practices; and the child's lifestyle and health include:
  ‐ anticipation of the next developmental milestones of the child;
  ‐ beyond knowledge acquisition, the exploration of barriers and facilitators for behavioral change within a one‐on‐one care and by interacting with peers;
  ‐ engagement of mothers/parents in a trustful, respectful and non‐judgmental guidance;
  ‐ the setting of individualized, gradual and achievable goals;
  ‐ the improvement of reflexivity and responsiveness to infant hunger and fullness cues;
  ‐ and parent modelling of healthy behaviors;
- in addition to the frequent focus on individual agency towards behavior change, it is important to help parents experiencing socio‐economic disadvantage identify and use the various resources available at the local level, which will foster their empowerment and navigation through parenthood with increased latitude and self‐efficacy;
- home‐visiting is suited to reach families experiencing disadvantage, but health‐care center based interventions were proven effective too, as long as a multidisciplinary team was involved;
- social support is also enhanced when group sessions with peers complement one‐on‐one support;
- when ethnic/racial minority groups are targeted, lay support, bilingual delivery agents, and culturally sensitive tools, improve inclusion and relevance.

### 4.5 Recommendations for future interventional research

The current systematic review revealed some gaps. Future interventional research should:

- use participatory approaches to better adapt programs to the recipients' needs and increase later transferability with stakeholders; and/or translate evidence‐based and theoretically underpinned programs;
- implement strategies in the trial design for increasing reach and retention (e.g. incentives, gifts, newsletters, repeated phone calls, travels reimbursed, as was done in part of the interventions reviewed here^62^, ^67^, ^68^, ^69^, ^70^, ^73^, ^74^, ^75^, ^76^, ^77^, ^78^, ^79^, ^80^, ^81^, ^82^, ^83^, ^84^, ^85^, ^86^, ^87^, ^88^, ^89^, ^90^, ^91^), particularly challenging in hard‐to‐survey populations. This is of paramount importance for quality and generalization of the findings;
- further target and engage fathers, or any other trust person, who are a potential support of the mothers;
- further strengthen the focus on smoking prevention during pregnancy and the child sleep routines in multi‐behavioral interventions, as they have been relatively neglected so far.^49^, ^51^, ^102^
- in addition to tailored counselling and advices, further support families experiencing disadvantage with structural facilitators, such as incentives, vouchers, food stamps, or coupons to facilitate healthy EBRBs^4^;
- further assess the use of social media and digitally based delivery modes^43^, ^52^, ^103^;
- assess the kinetics of early growth, along with measurements accounting for body composition, complementary to punctual measurements of weight, height and BMI; and, if feasible, measure the impact of interventions on anthropometrics at later follow‐ups, after the age at adiposity rebound, to better account for the long‐term and cumulative effect of unhealthy versus healthy behavioral trajectories. When it comes to EBRBs measurements, objective assessments are to be prioritized to increase precision and accuracy (e.g. accelerometry for physical activity and sedentary times). When this is not feasible, due to the behavior targeted (e.g. feeding practices and diet), validated and reproducible questionnaires or tools would improve internal validity of findings.^43^
- undertake moderation and mediation analyzes to better disentangle the contexts and mechanisms of action;
- use methods mixing quantitative and qualitative assessments, which are relevant^131^, ^132^:
  ‐ in pilot studies, to refine the various components of interventions, and increase their feasibility and acceptability, prior to the main trial implementation;
  ‐ to maximize the screening and recruitment processes, along with fidelity to the trial;
  ‐ to address process evaluation more comprehensively;
- detail the various items contributing to both internal and external validities more systematically, while reporting the findings of interventions, as all these elements are essential for their replication.^53^

## 5 CONCLUSION

Even though internal and external validity of most of these 24 interventions could be improved, the current review suggests some effectiveness on behavioral and anthropometric outcomes in young children when programs target and are tailored to families experiencing socio‐economic disadvantage. This is worth consideration by practitioners, researchers and policy makers given that parents facing social adversities are the most at‐risk for suboptimal lifestyle and health trajectories, along with their transmission to the next generation. In all, the most effective early OW/OB prevention interventions targeting underserved families are likely those that assemble the three types of interventions identified in the present review, i.e. broad parent support programs implemented over the first 1,000 days (pregnancy included) to optimize the physical and psycho‐social health of the mother and the mother–child bonding, along with an additional focus on BF, responsive feeding practices, and the various EBRBs. The latter should include the reduction of screen time and energy‐dense diets, along with the promotion of movement, active play, nutrient‐dense diet and sleep routines. Co‐creation with stakeholders, including parents; adherence to theoretical frameworks; engagement of multi‐disciplinary teams, including lay agents, along with groups of peers; are ingredients for more pragmatic, meaningful, inclusive, and non‐judgmental actions. Furthermore, a range of recommendations for future research and practice have been made, including a better engagement of fathers/partners to support the mother/child dyad, an increased focus on the prevention of smoking during pregnancy, and a more comprehensive support of families experiencing disadvantage with structural facilitators, using systems changes across multiple sectors and settings, to synergistically enable healthy behaviors. Only such holistic, multilevel, and proportionated interventions, are likely to more effectively and sustainably address the issue of social inequalities and inequities. More thorough process evaluation of such complex interventions using mixed methods is also needed to better understand why a given intervention worked or did not, by which mechanisms of action (if any), for whom and in which context. All these elements are essential for scaling‐up and translating effective programs into the routine practice.

## AUTHOR CONTRIBUTIONS

Sandrine Lioret and Rachel Laws conceived the review. Sandrine Lioret had the primary responsibility of preparing and conducting the search. Céline Van Baaren did preliminary searches during her master internship. Sandrine Lioret and Faryal Harrar then finalized the search strategy and formally screened all references and extracted the data. Sandrine Lioret analyzed the results and drafted the manuscript. **Kylie D. Hesketh and Rachel Laws provided their expertise having authored previous systematic reviews on similar topics.** All co‐authors were involved in revising the manuscript and reviewing the final version. All authors approved the final manuscript.

## CONFLICT OF INTEREST

No conflict of interest statement.

## Supporting information


**Table S1:** Internal validity: risk‐of‐bias (RoB) assessed by domains and overall, for each set of outcomes, for each intervention (Sterne et al, 2019)
**Table S2a**: External validity component 1 – Reach and representativeness of individuals
**Table S2b**: External validity component 2 – Reach and representativeness of settings
**Table S2c**: External validity component 3 – Implementation and adaptation
**Table S2d**: External validity component 4 – Outcomes for decision makers, maintenance and institutionalization
