# Supplementary material for: The effectiveness of interventions during the first 1,000 days to improve energy balance‐related behaviors or prevent overweight/obesity in children from socio‐economically disadvantaged families of high‐income countries: a systematic review
Source: Obes Rev. 2022 Nov 17;24(1):e13524. doi: 10.1111/obr.13524 (PMC10078443; doi:10.1111/obr.13524)
Supplement: Supplementary file 1 — Table S1: Internal validity: risk‐of‐bias (RoB) assessed by domains and overall, for each set of outcomes, for each intervention (Sterne et al, 2019) Table S2a: External validity component 1 – Reach and representativeness of individuals Table S2b: External validity component 2 – Reach and representativeness of settings Table S2c: External validity component 3 – Implementation and adaptation Table S2d: External validity component 4 – Outcomes for decision makers, maintenance and institutionalization [file OBR-24-0-s001.pdf]

Title: The effectiveness of interventions during the first 1000 days to improve energy balance-related behaviors or prevent overweight/obesity in children from socio-economically disadvantaged families of high-income countries: a systematic review

Authors: Sandrine Lioret[1], Faryal Harrar[1], Delia Boccia[2], Kylie D Hesketh[3], Konsita Kuswara[4], Céline Van Baaren[1], Silvia Maritano[4], Marie-Aline Charles[1], Barbara Heude[1], Rachel Laws[3]

[1]Université Paris Cité, INSERM, INRAE, CRESS, Paris, France
[2] Faculty of Public Health and Policy, Department of Global Health and Development, London School of Hygiene and Tropical Medicine, London, UK
[3] Deakin University, Institute for Physical Activity and Nutrition, School of Exercise and Nutrition Science, Geelong Australia
[4] Università di Torino, Cancer Epidemiology Unit, Department of Medical Sciences, Turin, Italy

Corresponding author: Sandrine Lioret
INSERM CRESS —Eq6 EAROH,
16 avenue Paul Vaillant Couturier,
94807 Villejuif Cedex, France.
Tel.: +33 1 45 59 51 78.
E-mail: sandrine.lioret@inserm.fr
ORCID : 0000-0002-2483-7820

Supplementary document S1: PRISMA checklist

Section and Topic	Item #	Checklist item	Location where item is reported
**TITLE**			
Title	1	Identify the report as a systematic review.	**Title**, in the Title page
**ABSTRACT**			
Abstract	2	See the PRISMA 2020 for Abstracts checklist.	**Abstract**, page 1
**INTRODUCTION**			
Rationale	3	Describe the rationale for the review in the context of existing knowledge.	Pages 2 and 3 (**Introduction** section)
Objectives	4	Provide an explicit statement of the objective(s) or question(s) the review addresses.	Page 2 (end of **Introduction** section)
**METHODS**			
Eligibility criteria	5	Specify the inclusion and exclusion criteria for the review and how studies were grouped for the syntheses.	Pages 3 and 4 (**2.1. Study selection criteria** section) and Table 1 (PICOT framework)
Information sources	6	Specify all databases, registers, websites, organisations, reference lists and other sources searched or consulted to identify studies. Specify the date when each source was last searched or consulted.	Pages 4 and 5 (**2.2 Search** section)
Search strategy	7	Present the full search strategies for all databases, registers and websites, including any filters and limits used.	Supplementary document S2
Selection process	8	Specify the methods used to decide whether a study met the inclusion criteria of the review, including how many reviewers screened each record and each report retrieved, whether they worked independently, and if applicable, details of automation tools used in the process.	Page 5 (**2.3. Study selection** section)
Data collection process	9	Specify the methods used to collect data from reports, including how many reviewers collected data from each report, whether they worked independently, any processes for obtaining or confirming data from study investigators, and if applicable, details of automation tools used in the process.	Page 5 (**2.4. Data extraction** section)
Data items	10a	List and define all outcomes for which data were sought. Specify whether all results that were compatible with each outcome domain in each study were sought (e.g. for all measures, time points, analyses), and if not, the methods used to decide which results to collect.	Page 5 (**2.4. Data extraction** section) and Table 1

Section and Topic	Item #	Checklist item	Location where item is reported
	10b	List and define all other variables for which data were sought (e.g. participant and intervention characteristics, funding sources). Describe any assumptions made about any missing or unclear information.	Page 5 (**2.4. Data extraction** section)
Study risk of bias assessment	11	Specify the methods used to assess risk of bias in the included studies, including details of the tool(s) used, how many reviewers assessed each study and whether they worked independently, and if applicable, details of automation tools used in the process.	Pages 5 and 6 (**2.5. Quality assessment** section)
Effect measures	12	Specify for each outcome the effect measure(s) (e.g. risk ratio, mean difference) used in the synthesis or presentation of results.	NA (narrative review, see page 6, **2.6. Synthesis of results** section)
Synthesis methods	13a	Describe the processes used to decide which studies were eligible for each synthesis (e.g. tabulating the study intervention characteristics and comparing against the planned groups for each synthesis (item #5)).	
	13b	Describe any methods required to prepare the data for presentation or synthesis, such as handling of missing summary statistics, or data conversions.	
	13c	Describe any methods used to tabulate or visually display results of individual studies and syntheses.	
	13d	Describe any methods used to synthesize results and provide a rationale for the choice(s). If meta-analysis was performed, describe the model(s), method(s) to identify the presence and extent of statistical heterogeneity, and software package(s) used.	
	13e	Describe any methods used to explore possible causes of heterogeneity among study results (e.g. subgroup analysis, meta-regression).	
	13f	Describe any sensitivity analyses conducted to assess robustness of the synthesized results.	
Reporting bias assessment	14	Describe any methods used to assess risk of bias due to missing results in a synthesis (arising from reporting biases).	Pages 5 and 6 (**2.5. Quality assessment** section)
Certainty assessment	15	Describe any methods used to assess certainty (or confidence) in the body of evidence for an outcome.	Pages 5 and 6 (**2.5. Quality assessment** section)
**RESULTS**			
Study selection	16a	Describe the results of the search and selection process, from the number of records identified in the search to the number of studies included in the review, ideally using a flow diagram.	Pages 6 and 7 (**3.1. Identification of studies** section) and **Figure 1.** PRISMA flow chart resulting from the search strategy
	16b	Cite studies that might appear to meet the inclusion criteria, but which were excluded, and explain why they were excluded.	**Figure 1.** PRISMA flow chart resulting from the search strategy
Study characteristics	17	Cite each included study and present its characteristics.	Pages 7-11 (**3.2. Study characteristics** and **3.3. Interventions' characteristics** sections) and Table 2
Risk of bias in studies	18	Present assessments of risk of bias for each included study.	Table S1
Results of individual studies	19	For all outcomes, present, for each study: (a) summary statistics for each group (where appropriate) and (b) an effect estimate and its precision (e.g. confidence/credible interval), ideally using structured tables or plots.	Pages 11-13 (**3.4. Impact on outcomes** section) and Tables 3 and 4
Results of syntheses	20a	For each synthesis, briefly summarise the characteristics and risk of bias among contributing studies.	
	20b	Present results of all statistical syntheses conducted. If meta-analysis was done, present for each the summary estimate and its precision (e.g. confidence/credible interval) and measures of statistical heterogeneity. If comparing groups, describe the direction of the effect.	NA (narrative review)
	20c	Present results of all investigations of possible causes of heterogeneity among study results.	
	20d	Present results of all sensitivity analyses conducted to assess the robustness of the synthesized results.	

Section and Topic	Item #	Checklist item	Location where item is reported
Reporting biases	21	Present assessments of risk of bias due to missing results (arising from reporting biases) for each synthesis assessed.	Pages 13-15 (**3.5. Internal validity** and **3.6 External validity** sections) and Tables S1 and S2
Certainty of evidence	22	Present assessments of certainty (or confidence) in the body of evidence for each outcome assessed.	
**DISCUSSION**			
Discussion	23a	Provide a general interpretation of the results in the context of other evidence.	Pages 15-22
	23b	Discuss any limitations of the evidence included in the review.	Page 22 (**4.3. Limitations and strengths** section)
	23c	Discuss any limitations of the review processes used.	
	23d	Discuss implications of the results for practice, policy, and future research.	Pages 22-25 (sections **4.4.** and **4.5**.)
**OTHER INFORMATION**			
Registration and protocol	24a	Provide registration information for the review, including register name and registration number, or state that the review was not registered.	Registered on PROSPERO, Registration ID number CRD42020166483
	24b	Indicate where the review protocol can be accessed, or state that a protocol was not prepared.	Protocol available at the PROSPERO registration website
	24c	Describe and explain any amendments to information provided at registration or in the protocol.	
Support	25	Describe sources of financial or non-financial support for the review, and the role of the funders or sponsors in the review.	Page 27 (**Acknowledgment** section)
Competing interests	26	Declare any competing interests of review authors.	Page 26 (**Conflict of interest** section)
Availability of data, code and other materials	27	Report which of the following are publicly available and where they can be found: template data collection forms; data extracted from included studies; data used for all analyses; analytic code; any other materials used in the review.	Page 6, at the end of the **2.5.** section)

From: Page MJ, McKenzie JE, Bossuyt PM, Boutron I, Hoffmann TC, Mulrow CD, et al. The PRISMA 2020 statement: an updated guideline for reporting systematic reviews. BMJ 2021;372:n71. doi: 10.1136/bmj.n71

For more information, visit: http://www.prisma-statement.org/

Supplementary document S2: Search strategies: examples in PubMed/MEDLINE and Scopus

PubMed/MEDLINE

(((((infant[tiab] OR infants[tiab] OR infancy[tiab] OR pregnan*[tiab] OR newborn*[tiab] OR toddler*[tiab] OR baby[tiab] OR babies[tiab] OR mother*[tiab] OR father*[tiab] OR parent[tiab] OR parents[tiab] OR mum[tiab] OR mums[tiab] OR maternal[tiab] OR dad[tiab] OR dads[tiab] OR paternal[tiab] or mom[tiab] or moms[tiab] OR Infant[Mesh] OR Pregnancy[Mesh] OR Parents[Mesh])) AND (Emigrants and Immigrants[Mesh] OR Transients and Migrants[Mesh] OR Vulnerable Populations[Mesh] OR Refugees[Mesh] OR Ethnic Groups[Mesh] OR Homeless Persons[Mesh] OR Socioeconomic Factors[Mesh] OR Psychosocial Deprivation[Mesh] OR "adolescent mother"[tiab] OR "adolescent mothers"[tiab] OR wic[tiab] OR “low income” [tiab] OR "disadvantaged"[tiab] OR poverty[tiab] OR poor[tiab] or "poverty areas"[tiab] OR underprivileg*[tiab] OR unemploy*[tiab] OR nonemploy*[tiab] OR homeless[tiab] OR immigrant*[tiab] OR emigrant*[tiab] OR migrant*[tiab] OR "travelling community"[tiab] OR "travelling communities"[tiab] OR refugees[tiab]))

AND (Feeding Behavior[Mesh] OR Body Mass Index[Mesh] OR Body Size[Mesh] or skinfold thickness[Mesh] or Waist-hip ratio[Mesh] OR Diet[Mesh] OR Food and Beverages[Mesh] OR Infant Nutritional Physiological Phenomena[Mesh] OR Sedentary Behavior[Mesh] OR Play and Playthings[Mesh] OR Exercise[Mesh] OR Television[Mesh] OR overweight[tiab] or "weight gain"[tiab] or "weight loss"[tiab] OR "over weight"[tiab] OR

obesity[tiab] OR obesogenic[tiab] OR weight[tiab] OR height[tiab] OR BMI[tiab] OR "body mass index"[tiab] OR feeding[tiab] OR nutrition*[tiab] OR diet[tiab] OR fruit*[tiab] OR vegetable*[tiab] OR beverage*[tiab] OR drink*[tiab] OR soda*[tiab] OR tummy[tiab] OR "feeding behavior"[tiab] OR "feeding behaviour"[tiab] or “Feeding practices"[tiab] OR "food habit"[tiab] OR "food habits"[tiab] OR "eating habit"[tiab] OR "eating habits"[tiab] OR play [tiab] OR outdoor[tiab] OR "physical activity"[tiab] OR "physical activities"[tiab] OR sedentary[tiab] OR television[tiab] OR screen[tiab] OR movement[tiab] OR "motor activity"[tiab] OR accelerometry[tiab] OR TV[tiab] or weaning[tiab] or breastfeeding[tiab] OR "video game"[tiab] OR "video games"[tiab] OR inactiv*[tiab] OR digital tablet*[tiab] OR exercise*[tiab] OR “tobacco smoking” [mesh] or “tobacco smoking” [tiab] or “tobacco”[tiab] or "cigarette"[tiab]))

AND (Community Health Services[Mesh] OR Preventive Health Services[Mesh] OR Parenting[Mesh] OR Health Knowledge, Attitudes, Practice[Mesh] OR Infant care[Mesh] OR Health Behavior[Mesh] OR Cognitive Behavioral Therapy[Mesh] OR Diet therapy[Mesh] OR Exercise therapy[Mesh] OR Motivation[Mesh] OR Social Support[Mesh] OR "home visiting"[tiab] OR “Early Intervention”[tiab] OR intervention[tiab] OR prevention[tiab] OR parenting[tiab] OR programme[tiab] OR program[tiab] OR counseling[tiab] OR "social support"[tiab] OR motivat*[tiab] OR "parent group"[tiab] OR "mother group"[tiab] OR "parent groups"[tiab] OR "mother groups"[tiab]))

AND (Clinical study[ptyp] OR Comparative Study[ptyp] OR Evaluation Studies[ptyp])

<u>Scopus</u>

TITLE-ABS-KEY(toddler* or babies or baby or infan* or parent* or mother* or father* or mum* or dad* or mom* or maternal or paternal or newborn* or pregnan* or preschool and unemploy* or nonemploy* or "non-employ*" or disadvant* or "social disadvantag*" or "socioeconomic disadvantag*" or "socio-economic disadvantag*" or "socially disadvantag*" or "food insecurity" or "adolescent mother*" or "wic" or underprivileg* or vulnerable* or "low income" or "low-income" or poverty or "poverty areas" or poor or immigrant* or emigrant* or migrant* or "travelling communit*" or transcient* or refugees or "ethnic group*" or "african american*" or "hispanic american*" or "mexican american*" or homeless and "physic* activ*" or exercis* or movement or "motor activ*" or tummy or accelerometry or outdoor or play* or television or tv or screen or "video game*" or sedentar* or inactiv* or obes* or overweight or "over weight" or "weight gain" or "weight loss" or bmi or "body mass index" or "body fat" or « height » or « weight » or adiposity or "skinfold thickness" or diet* or nutrition* or fruit* or vegetable* or beverage* or drink* or "energy intake*" or "dietary intake" or "dietary pattern*" or soda* or food* or "food habit*" or "eating habit" or "complementary food*" or "breast-feeding" or breastfeeding or "breast feeding" or "formula-feeding" or « bottle feeding » or weaning or "diet habit*" or "diet fat*" of "feeding practice*" or feeding or "feeding behavior*" or "feeding behaviour*" or smoking or tobacco or cigarette* and "social support" or "parent group*" or « infant care » or "mother group*" or counsel* or program* or "home visit*" or voucher* or "food stamp*" or incentive* or prevent* or "early intervention" or "child* obesity prevention" or "smoking reduction" or "behav* change*" or motivat* or "exercise therapy" or parenting and interven* or "clinical trial" or "randomi?ed control* trial" or rct or "comparative stud*" or "comparison stud*" or "cross over stud*" or "cross-over stud*" or "evaluation stud*") AND PUBYEAR > 1989

Table S1: Internal validity: risk-of-bias (RoB) assessed by domains and overall, for each set of outcomes, for each intervention (Sterne et al., 2019)

**Study reference**	**Outcomes**	**Domains**					**Overall RoB**
		1.RoB arising from the randomization process	2.RoB due to deviations from the intended interventions	3.Missing outcome data	4.RoB in measurement of the outcome	5.RoB in selection of the reported result	
(Wen et al., 2012)	Anthro						
	PFP; Diet; PA; SB						
(Wen et al., 2011)	BF; PFP; PA						
(Wen et al., 2015)	Anthro						
	PFP; Diet; PA; SB						
(Bonuck et al., 2014)	Anthro						
	PFP; Diet						
(Gross et al., 2016)	BF; PFP						
(Gross et al., 2017)	PA; SB						
(Messito et al., 2020a)	BF; PFP; Diet						
(Messito et al., 2020b)	Anthro						
(Fiks et al., 2017)	Anthro						
	BF; PFP; PA; SB; Sleep						
(Reifsnider et al., 2018)	Anthro						
	BF						
	PFP						
(McCormick et al., 2020)	Anthro						
(Black et al., 2021)	PFP; Diet; PA; Anthro						
(Black et al., 2001)	PFP						
(Horodynski and Stommel, 2005)	PFP						
(Watt et al., 2009)	Anthro						
	BF; PFP						
	Diet						
(Scheiwe et al., 2010)	Anthro						
	PFP; Diet						
(Scheinmann et al., 2010)	BF; PFP						
(Edwards et al., 2013)	BF						
	PFP						
(Johnson et al., 1993)	PFP; Diet						
(Johnson et al., 2000)	Diet						
(Kitzman et al., 1997)	Anthro						
	BF						

Study reference	Outcomes	Domains: 1.RoB arising from the randomization process	2.RoB due to deviations from the intended interventions	3.Missing outcome data	4.RoB in measurement of the outcome	5.RoB in selection of the reported result	Overall RoB
(Alvarado et al., 1999)	Anthro	High	Some concerns	Low	High	Some concerns	High
	BF	High	Some concerns	Low	High	Some concerns	High
(Wiggins et al., 2005)	BF; PFP	Low	Some concerns	Low	Low	Low	Some concerns
(Cupples et al., 2011)	BF; PA; Anthro	Low	Some concerns	Low	Low	Low	Some concerns
(Kemp et al., 2011)	Anthro	Low	Some concerns	Low	Low	Low	Some concerns
	BF; PFP	Low	Some concerns	Low	Some concerns	Low	Some concerns
	PA	Low	Some concerns	Some concerns	Low	Low	Some concerns
(Mejdoubi et al., 2014)	Anthro	Some concerns	Some concerns	Low	Low	Low	Some concerns
	BF	Some concerns	Some concerns	Low	Low	Low	Some concerns
(Kenyon et al., 2016)	Anthro	Low	Some concerns	Low	Low	Low	Some concerns
	BF	Low	Some concerns	Low	Low	Low	Some concerns
(Popo et al., 2017)	Anthro	Low	Some concerns	Some concerns	Low	Some concerns	Some concerns
	PA	Low	Some concerns	Some concerns	Low	Some concerns	Some concerns
(O'Sullivan et al., 2017)	Diet	Low	Some concerns	Low	Low	Low	Some concerns
(Ordway et al., 2018)	Anthro	High	Some concerns	Some concerns	Low	Some concerns	High
(Hans et al., 2018a, b)	Anthro	Low	Some concerns	Low	Some concerns	High	High
	BF	Low	Some concerns	Low	Low	Low	Some concerns
(Lutenbacher et al., 2018a, b)	BF; PFP	Some concerns	Some concerns	Low	Low	Some concerns	Some concerns
(Goldfeld et al., 2019)	BF; PFP; Diet; Sleep	Low	Some concerns	Low	Low	Low	Some concerns

Abbreviations: **BF,** breastfeeding; **PFP,** Parental feeding practices other than BF; **PA,** physical activity; **SB,** sedentary behavior; **Anthro,** anthropometrics; **SL,** sleep.

Risk-of-bias judgement: Low risk of bias; some concerns; high risk of bias

Supplementary Tables S2

Table S2a: External validity component 1 – Reach and representativeness of individuals

	Target population described	Methods to recruit target population described	Individual inclusion and exclusion reported	Enrolment rate	Recruitment rate	Representativeness of participants described
(Wen et al., 2011; Wen et al., 2012; Wen et al., 2015)	Y	Y	Y	Y	Y	N
(Bonuck et al., 2014)	Y	Y	Y	Y	N	N
(Gross et al., 2016; Gross et al., 2017; Messito et al., 2020a; Messito et al., 2020b)	Y	Y	Y	Y	Y	N
(Fiks et al., 2017)	Y	Y	Y	Y	Y	N
(McCormick et al., 2020; Reifsnider et al., 2018)	Y	Y	Y	Y	Y	N
(Black et al., 2021)	Y	N	Y	Y	Y	N
(Black et al., 2001)	Y	Y	Y	Y	N	Y
(Horodynski and Stommel, 2005)	Y	Y	Y	N	N	N
(Scheiwe et al., 2010; Watt et al., 2009)	Y	Y	Y	Y	Y	Y
(Scheinmann et al., 2010)	Y	Y	Y	N	N	N
(Edwards et al., 2013)	Y	Y	Y	N	Y	N
(Johnson et al., 1993; Johnson et al., 2000)	Y	Y	N	Y	Y	N
(Kitzman et al., 1997)	Y	Y	N	Y	Y	Y
(Alvarado et al., 1999)	Y	Y	N	Y	N	N
(Wiggins et al., 2005)	Y	Y	Y	Y	Y	Y
(Cupples et al., 2011)	Y	Y	Y	Y	Y	Y
(Mejdoubi et al., 2014)	Y	Y	Y	Y	Y	N
(Kenyon et al., 2016; Popo et al., 2017)	Y	Y	Y	N	Y	N
(Doyle et al., 2014; O'Sullivan et al., 2017)	Y	Y	Y	Y	Y	N
(Ordway et al., 2018)	Y	Y	Y	Y	Y	N
(Hans et al., 2018a, b)	Y	Y	Y	Y	Y	N
(Lutenbacher et al., 2018a, b)	Y	Y	Y	Y	Y	N
(Goldfeld et al., 2019; Kemp et al., 2011)[a]	Y	Y	Y	Y	Y	Y

Abbreviations: **Y,** yes; **N,** no; **NI,** no information; **NA,** not applicable.

[a]The Right@Home intervention (Goldfeld et al., 2019) is the translation of the Maternal Early Childhood Sustained Home-visiting (MECSH) program (Kemp et al., 2011), thus these two interventions were considered together for external validity assessment.

Table S2b: External validity component 2 – Reach and representativeness of settings

	Target setting described	Methods to recruit target setting described	Setting inclusion and exclusion reported	Setting participation rate	Representativeness of settings described
(Wen et al., 2011; Wen et al., 2012; Wen et al., 2015)	Y	N	N	N	N
(Bonuck et al., 2014)	Y	Y	N	N	N
(Gross et al., 2016; Gross et al., 2017; Messito et al., 2020a; Messito et al., 2020b)	Y	N	N	N	N
(Fiks et al., 2017)	Y	N	N	N	N
(McCormick et al., 2020; Reifsnider et al., 2018)	Y	N	Y	N	N
(Black et al., 2021)	Y	N	N	N	N
(Black et al., 2001)	Y	N	N	N	N
(Horodynski and Stommel, 2005)	Y	N	N	N	N
(Scheiwe et al., 2010; Watt et al., 2009)	Y	N	N	N	N
(Scheinmann et al., 2010)	Y	N	N	N	N
(Edwards et al., 2013)	Y	N	N	N	N
(Johnson et al., 1993; Johnson et al., 2000)	Y	N	N	N	N
(Kitzman et al., 1997)	Y	N	N	N	N
(Alvarado et al., 1999)	Y	N	N	N	N
(Wiggins et al., 2005)	Y	N	Y	N	N
(Cupples et al., 2011)	Y	N	N	N	N
(Mejdoubi et al., 2014)	Y	N	N	N	N
(Kenyon et al., 2016; Popo et al., 2017)	Y	Y	N	N	N
(Doyle et al., 2014; O'Sullivan et al., 2017)	Y	N	N	N	N
(Ordway et al., 2018)	Y	N	N	N	N
(Hans et al., 2018a, b)	Y	Y	N	N	N
(Lutenbacher et al., 2018a, b)	Y	N	N	N	N
(Goldfeld et al., 2019; Kemp et al., 2011)[a]	Y	Y	N	N	N

Abbreviations: **Y,** yes; **N,** no; **NI,** no information; **NA,** not applicable.

[a]The Right@Home intervention (Goldfeld et al., 2019) is the translation of the Maternal Early Childhood Sustained Home-visiting (MECSH) program (Kemp et al., 2011), thus these two interventions were considered together for external validity assessment.

Table S2c: External validity component 3 – Implementation and adaptation

	Intervention characteristics described	Time to deliver intervention described	Intervention exposure reported	Delivery agent described	Methods to recruit delivery agents described	Delivery agents participation rate described	Training of delivery agent described	Intervention fidelity measured	Mechanisms for intervention effects
(Wen et al., 2011; Wen et al., 2012; Wen et al., 2015)	Y	Y	Y	Y	N	N	N	N	N
(Bonuck et al., 2014)	Y	Y	N	Y	Y	N	Y	N	N
(Gross et al., 2016; Gross et al., 2017; Messito et al., 2020a; Messito et al., 2020b)	Y	Y	Y	Y	N	N	Y	Y	Y
(Fiks et al., 2017)	Y	Y	Y	Y	N	N	N	Y	N
(McCormick et al., 2020; Reifsnider et al., 2018)	Y	N	N	Y	Y	N	Y	N	N
(Black et al., 2021)	Y	N	Y	Y	Y	N	Y	Y	N
(Black et al., 2001)	Y	N	N	Y	N	N	Y	N	N
(Horodynski and Stommel, 2005)	Y	Y	Y	Y	N	N	N	Y	N
(Scheiwe et al., 2010; Watt et al., 2009)	Y	Y	Y	Y	Y	Y	Y	N	N
(Scheinmann et al., 2010)	Y	Y	Y	Y	N	N	NA (video)	N	N
(Edwards et al., 2013)	Y	N	Y	Y	N	N	Y	N	N
(Johnson et al., 1993; Johnson et al., 2000)	Y	N	Y	Y	Y	N	Y	N	N
(Kitzman et al., 1997)	Y	Y	Y	Y	Y	N	N	N	N
(Alvarado et al., 1999)	Y	N	Y	Y	Y	N	Y	N	N
(Wiggins et al., 2005)	Y	Y	Y	Y	Y	N	Y	N	N
(Cupples et al., 2011)	Y	N	Y	Y	Y	Y	Y	N	N
(Mejdoubi et al., 2014)	Y	Y	Y	Y	Y	N	Y	N	N
(Kenyon et al., 2016; Popo et al., 2017)	Y	Y	Y	Y	Y	N	Y	Y	N
(Doyle et al., 2014; O'Sullivan et al., 2017)	Y	Y	Y	Y	Y	N	Y	Y	Y
(Ordway et al., 2018)	Y	Y	Y	Y	Y	N	Y	N	N
(Hans et al., 2018a, b)	Y	N	Y	Y	N	N	Y	N	N
(Lutenbacher et al., 2018a, b)	Y	Y	N	Y	Y	N	Y	Y	N
(Goldfeld et al., 2019; Kemp et al., 2011)[a]	Y	Y	Y	Y	Y	N	Y	Y	N

Abbreviations: **Y,** yes; **N,** no; **NI,** no information; **NA,** not applicable.

[a]The Right@Home intervention (Goldfeld et al., 2019) is the translation of the Maternal Early Childhood Sustained Home-visiting (MECSH) program (Kemp et al., 2011), thus these two interventions were considered together for external validity assessment.

Table S2d: External validity component 4 – Outcomes for decision makers, maintenance and institutionalization

	Outcomes compared to standards	Adverse conse-quences reported	Effect moderator by participant characte-ristics	Effect moderator by setting/ delivery agent	Dose response effect of the interven-tion	Interven-tion costs or cost effective-ness	Attrition rates reported	Differen-tial attrition rates reported	Represen-tativeness of completers/ drop-outs	Long term effects (>6 months)[a]	Accepta-bility	Institu-tionaliza-tion
(Wen et al., 2011; Wen et al., 2012; Wen et al., 2015)	Y	N	Y	N	N	Y	Y	Y	Y	Y	Y	N
(Bonuck et al., 2014)	Y	N	N	N	N	N	Y	Y	NI	N	Y	Y
(Gross et al., 2016; Gross et al., 2017; Messito et al., 2020a; Messito et al., 2020b)	Y	Y	Y	N	Y	N	Y	Y	Y	N	N	Y
(Fiks et al., 2017)	Y	N	N	N	N	N	Y	Y	Y	N	Y	N
(McCormick et al., 2020; Reifsnider et al., 2018)	Y	Y	N	N	N	N	Y	Y	Y	Y	N	N
(Black et al., 2021)	Y	Y	Y	N	Y	N	Y	Y	N	Y	N	N
(Black et al., 2001)	Y	N	N	N	N	N	Y	Y	N	N	N	N
(Horodynski and Stommel, 2005)	Y	N	N	N	N	N	Y	Y	NI	N	Y	N
(Scheiwe et al., 2010; Watt et al., 2009)	Y	N	N	N	N	N	Y	Y	NI	Y	Y	N
(Scheinmann et al., 2010)	Y	N	N	N	N	N	Y	Y	NI	N	N	N
(Edwards et al., 2013)	Y	N	N	N	N	N	Y	Y	NI	N	Y	N
(Johnson et al., 1993; Johnson et al., 2000)	Y	N	N	N	N	Y	Y	Y	N	Y	N	N
(Kitzman et al., 1997)	Y	N	N	N	Y	Y	Y	Y	N	N	N	Y
(Alvarado et al., 1999)	Y	N	N	N	N	N	Y	Y	Y	N	Y	N
(Wiggins et al., 2005)	Y	N	Y	Y	Y	Y	Y	Y	Y	N	Y	Y
(Cupples et al., 2011)	Y	N	N	N	N	N	Y	Y	NI	N	Y	N
(Mejdoubi et al., 2014)	Y	N	Y	N	N	N	Y	Y	N	N	Y	Y
(Kenyon et al., 2016; Popo et al., 2017)	Y	N	Y	N	N	Y	Y	Y	Y	N	N	N
(Doyle et al., 2014; O'Sullivan et al., 2017)	Y	N	N	N	N	N	Y	Y	Y	N	Y	N
(Ordway et al., 2018)	Y	N	Y	N	N	N	Y	Y	Y	N	Y	Y
(Hans et al., 2018a, b)	Y	N	N	N	N	N	Y	Y	N	N	Y	Y
(Lutenbacher et al., 2018a, b)	Y	N	N	N	N	Y	Y	Y	NI	N	Y	Y
(Goldfeld et al., 2019; Kemp et al., 2011)[b]	Y	N	Y	N	Y	Y	N	Y	Y	N	Y	Y

Abbreviations: **Y,** yes; **N,** no; **NI,** no information; **NA,** not applicable.

[a]Only outcomes eligible for the current review are considered here. [b]The Right@Home intervention (Goldfeld et al., 2019) is the translation of the Maternal Early Childhood Sustained Home-visiting (MECSH) program (Kemp et al., 2011), thus these two interventions were considered together for external validity assessment.

References

Alvarado, R., Zepeda, A., Rivero, S., Rico, N., Lopez, S., Diaz, S., 1999. Integrated maternal and infant health care in the postpartum period in a poor neighborhood in Santiago, Chile. Stud Fam Plann 30:133-41.

Black, M.M., Hager, E.R., Wang, Y., Hurley, K.M., Latta, L.W., Candelaria, M., Caulfield, L.E., 2021. Toddler obesity prevention: A two-generation randomized attention-controlled trial. Matern Child Nutr 17:e13075.

Black, M.M., Siegel, E.H., Abel, Y., Bentley, M.E., 2001. Home and videotape intervention delays early complementary feeding among adolescent mothers. Pediatrics 107:E67.

Bonuck, K., Avraham, S.B., Lo, Y., Kahn, R., Hyden, C., 2014. Bottle-weaning intervention and toddler overweight. J Pediatr 164:306-12 e1-2.

Cupples, M.E., Stewart, M.C., Percy, A., Hepper, P., Murphy, C., Halliday, H.L., 2011. A RCT of peer-mentoring for first-time mothers in socially disadvantaged areas (the MOMENTS Study). Arch Dis Child 96:252-8.

Edwards, R.C., Thullen, M.J., Korfmacher, J., Lantos, J.D., Henson, L.G., Hans, S.L., 2013. Breastfeeding and complementary food: randomized trial of community doula home visiting. Pediatrics 132 Suppl 2:S160-6.

Fiks, A.G., Gruver, R.S., Bishop-Gilyard, C.T., Shults, J., Virudachalam, S., Suh, A.W., Gerdes, M., Kalra, G.K., DeRusso, P.A., et al., 2017. A Social Media Peer Group for Mothers To Prevent Obesity from Infancy: The Grow2Gether Randomized Trial. Child Obes 13:356-68.

Goldfeld, S., Price, A., Smith, C., Bruce, T., Bryson, H., Mensah, F., Orsini, F., Gold, L., Hiscock, H., et al., 2019. Nurse Home Visiting for Families Experiencing Adversity: A Randomized Trial. Pediatrics 143.

Gross, R.S., Mendelsohn, A.L., Gross, M.B., Scheinmann, R., Messito, M.J., 2016. Randomized Controlled Trial of a Primary Care-Based Child Obesity Prevention Intervention on Infant Feeding Practices. J Pediatr 174:171-77 e2.

Gross, R.S., Mendelsohn, A.L., Yin, H.S., Tomopoulos, S., Gross, M.B., Scheinmann, R., Messito, M.J., 2017. Randomized controlled trial of an early child obesity prevention intervention: Impacts on infant tummy time. Obesity (Silver Spring) 25:920-27.

Hans, S.L., Edwards, R.C., Zhang, Y., 2018a. Correction to: Randomized Controlled Trial of Doula-Home-Visiting Services: Impact on Maternal and Infant Health. Matern Child Health J 22:125.

Hans, S.L., Edwards, R.C., Zhang, Y., 2018b. Randomized Controlled Trial of Doula-Home-Visiting Services: Impact on Maternal and Infant Health. Matern Child Health J 22:105-13.

Horodynski, M.A., Stommel, M., 2005. Nutrition education aimed at toddlers: an intervention study. Pediatr Nurs 31:364, 67-72.

Johnson, Z., Howell, F., Molloy, B., 1993. Community mothers' programme: randomised controlled trial of non-professional intervention in parenting. BMJ 306:1449-52.

Johnson, Z., Molloy, B., Scallan, E., Fitzpatrick, P., Rooney, B., Keegan, T., Byrne, P., 2000. Community Mothers Programme--seven year follow-up of a randomized controlled trial of non-professional intervention in parenting. J Public Health Med 22:337-42.

Kemp, L., Harris, E., McMahon, C., Matthey, S., Vimpani, G., Anderson, T., Schmied, V., Aslam, H., Zapart, S., 2011. Child and family outcomes of a long-term nurse home visitation programme: a randomised controlled trial. Arch Dis Child 96:533-40.

Kenyon, S., Jolly, K., Hemming, K., Hope, L., Blissett, J., Dann, S.A., Lilford, R., MacArthur, C., 2016. Lay support for pregnant women with social risk: a randomised controlled trial. BMJ Open 6:e009203.

Kitzman, H., Olds, D.L., Henderson, C.R., Jr., Hanks, C., Cole, R., Tatelbaum, R., McConnochie, K.M., Sidora, K., Luckey, D.W., et al., 1997. Effect of prenatal and infancy home visitation by nurses on pregnancy outcomes, childhood injuries, and repeated childbearing. A randomized controlled trial. JAMA 278:644-52.

Lutenbacher, M., Elkins, T., Dietrich, M.S., Riggs, A., 2018a. Correction to: The Efficacy of Using Peer Mentors to Improve Maternal and Infant Health Outcomes in Hispanic Families: Findings from a Randomized Clinical Trial. Matern Child Health J 22:124.

Lutenbacher, M., Elkins, T., Dietrich, M.S., Riggs, A., 2018b. The Efficacy of Using Peer Mentors to Improve Maternal and Infant Health Outcomes in Hispanic Families: Findings from a Randomized Clinical Trial. Matern Child Health J 22:92-104.

McCormick, D.P., Reyna, L., Reifsnider, E., 2020. Calories, Caffeine and the Onset of Obesity in Young Children. Acad Pediatr 20:801-08.

Mejdoubi, J., van den Heijkant, S.C., van Leerdam, F.J., Crone, M., Crijnen, A., HiraSing, R.A., 2014. Effects of nurse home visitation on cigarette smoking, pregnancy outcomes and breastfeeding: a randomized controlled trial. Midwifery 30:688-95.

Messito, M.J., Katzow, M.W., Mendelsohn, A.L., Gross, R.S., 2020a. Starting Early Program Impacts on Feeding at Infant 10 Months Age: A Randomized Controlled Trial. Child Obes 16:S4-S13.

Messito, M.J., Mendelsohn, A.L., Katzow, M.W., Scott, M.A., Vandyousefi, S., Gross, R.S., 2020b. Prenatal and Pediatric Primary Care-Based Child Obesity Prevention Program: A Randomized Trial. Pediatrics 146.

O'Sullivan, A., Fitzpatrick, N., Doyle, O., 2017. Effects of early intervention on dietary intake and its mediating role on cognitive functioning: a randomised controlled trial. Public Health Nutr 20:154-64.

Ordway, M.R., Sadler, L.S., Holland, M.L., Slade, A., Close, N., Mayes, L.C., 2018. A Home Visiting Parenting Program and Child Obesity: A Randomized Trial. Pediatrics 141.

Popo, E., Kenyon, S., Dann, S.A., MacArthur, C., Blissett, J., 2017. Effects of lay support for pregnant women with social risk factors on infant development and maternal psychological health at 12 months postpartum. PLoS One 12:e0182544.

Reifsnider, E., McCormick, D.P., Cullen, K.W., Todd, M., Moramarco, M.W., Gallagher, M.R., Reyna, L., 2018. Randomized Controlled Trial to Prevent Infant Overweight in a High-Risk Population. Acad Pediatr 18:324-33.

Scheinmann, R., Chiasson, M.A., Hartel, D., Rosenberg, T.J., 2010. Evaluating a bilingual video to improve infant feeding knowledge and behavior among immigrant Latina mothers. J Community Health 35:464-70.

Scheiwe, A., Hardy, R., Watt, R.G., 2010. Four-year follow-up of a randomized controlled trial of a social support intervention on infant feeding practices. Matern Child Nutr 6:328-37.

Sterne, J.A.C., Savovic, J., Page, M.J., Elbers, R.G., Blencowe, N.S., Boutron, I., Cates, C.J., Cheng, H.Y., Corbett, M.S., et al., 2019. RoB 2: a revised tool for assessing risk of bias in randomised trials. BMJ 366:l4898.

Watt, R.G., Tull, K.I., Hardy, R., Wiggins, M., Kelly, Y., Molloy, B., Dowler, E., Apps, J., McGlone, P., 2009. Effectiveness of a social support intervention on infant feeding practices: randomised controlled trial. J Epidemiol Community Health 63:156-62.

Wen, L.M., Baur, L.A., Simpson, J.M., Rissel, C., Flood, V.M., 2011. Effectiveness of an early intervention on infant feeding practices and "tummy time": a randomized controlled trial. Arch Pediatr Adolesc Med 165:701-7.

Wen, L.M., Baur, L.A., Simpson, J.M., Rissel, C., Wardle, K., Flood, V.M., 2012. Effectiveness of home based early intervention on children's BMI at age 2: randomised controlled trial. BMJ 344:e3732.

Wen, L.M., Baur, L.A., Simpson, J.M., Xu, H., Hayes, A.J., Hardy, L.L., Williams, M., Rissel, C., 2015. Sustainability of Effects of an Early Childhood Obesity Prevention Trial Over Time: A Further 3-Year Follow-up of the Healthy Beginnings Trial. JAMA Pediatr 169:543-51.

Wiggins, M., Oakley, A., Roberts, I., Turner, H., Rajan, L., Austerberry, H., Mujica, R., Mugford, M., Barker, M., 2005. Postnatal support for mothers living in disadvantaged inner city areas: a randomised controlled trial. J Epidemiol Community Health 59:288-95.
